# Overview of renicolid digeneans (Digenea, Renicolidae) from marine gulls of northern Holarctic with remarks on their species statuses, phylogeny and phylogeography

**DOI:** 10.1017/S0031182022001500

**Published:** 2023-01

**Authors:** Kirill V. Galaktionov, Anna I. Solovyeva, April M. H. Blakeslee, Karl Skírnisson

**Affiliations:** 1 Laboratory of Parasitic Worms and Protists, Zoological Institute of Russian Academy of Sciences, St. Petersburg 199034, Russia; 2 Laboratory of Non-Coding DNA, Institute of Cytology of Russian Academy of Sciences, St. Petersburg 194064, Russia; 3 Department of Biology, East Carolina University, Greenville, NC, USA; 4 Marine Invasions Lab, Smithsonian Environmental Research Center, Edgewater, MD, USA; 5 Laboratory of Parasitology, Institute for Experimental Pathology, University of Iceland, Keldur, Reykjavik, Iceland

**Keywords:** Life cycle, *Littorina*, marine gulls, molecular phylogeny, phylogeography, *Renicola keimahuri*, *Renicola parvicaudatus*, Renicolidae, Rhodometopa cercariae

## Abstract

Renicolid digeneans parasitize aquatic birds. Their intramolluscan stages develop in marine and brackish-water gastropods, while metacercariae develop in molluscs and fishes. The systematics of renicolids is poorly developed, their life cycles are mostly unknown, and the statuses of many species require revision. Here, we establish based on integrated morphological and molecular data that adult renicolids from gulls *Larus argentatus* and *Larus schistisagus* and sporocysts and cercariae of *Cercaria parvicaudata* from marine snails *Littorina* spp. are life-cycle stages of the same species. We name it *Renicola parvicaudatus* and synonymized with it *Renicola roscovitus*. An analysis of the *cox1* gene of *R. parvicaudatus* from Europe, North America and North Asia demonstrates a low genetic divergence, suggesting that this species has formed quite recently (perhaps during last glacial maximum) and that interregional gene flow is high. In *Littorina saxatilis* and *L. obtusata* from the Barents Sea, molecular analysis has revealed intramolluscan stages of *Cercaria littorinae saxatilis* VIII, a cryptic species relative to *R. parvicaudatus*. In the molecular trees, *Renicola keimahuri* from *L. schistisagus* belongs to another clade than *R. parvicaudatus*. We show that the species of this clade have cercariae of Rhodometopa group and outline morphological and behavioural transformations leading from xiphidiocercariae to these larvae. Molecular analysis has revealed 3 main phylogenetic branches of renicolids, differing in structure of adults, type of cercariae and host range. Our results elucidate the patterns of host colonization and geographical expansion of renicolids and pave the way to the solution of some long-standing problems of their classification.

## Graphical abstract

**Figure:**
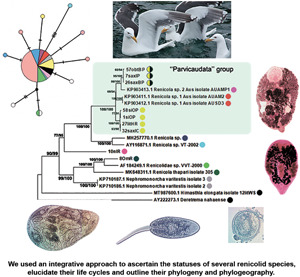

## Introduction

Renicolidae is a small family of digeneans (Trematoda, Digenea), currently comprising fewer than a hundred species, taking into account the descriptions of the larvae (Sudarikov and Stenko, 1984; Munyer and Holloway, 1990; Kharoo, 2013). Their transmission is implemented in marine and estuarine ecosystems. In the complex life cycle of renicolids, the role of the first intermediate host is played by marine and brackish-water gastropods, while the role of the second intermediate host is mostly played by molluscs and fish. Adult renicolids parasitize kidneys and ureters of marine or aquatic birds, exhibiting a strong pathogenic effect on their hosts (Campbell and Sloan, 1943; Hill, 1952, 1954; Riley and Owen, 1972; Mahdy and Shaheed, 2001; Jerdy *et al*., 2016; Matos *et al*., 2021). As they grow, their body becomes densely packed with eggs. It is next to impossible to discern diagnostic characters in such a worm. Considering, in addition, that adult worms of closely related species are similar morphologically, differentiating among them is a challenge. It is therefore unsurprising that the systematics of renicolids is poorly developed. Only 2 genera are recognized within the family: *Renicola* Cohn, 1904 and *Nephromonorcha* Leonov, 1958. Their adults differ in the number of testes: 2 separate testes in the former and 1 testis (resulting from merging of the 2) in the latter genus (Sudarikov and Stenko, 1984; Gibson, 2008). Attempts to elaborate the classification of renicolids (e.g. Wright, 1957; Odening, 1962; Riley and Owen, 1972) have not gained general recognition (reviewed in Gibson, 2008; Kharoo, 2013).

Intramolluscan stages of renicolids are represented by mother sporocysts, which look like small membrane-enveloped aggregations of cells, and cercariae-producing sac-like daughter sporocysts, parasitizing the molluscan gonad and digestive gland (Wright, 1956; James, 1969). Life cycles of only a few renicolid species have been elucidated (Stunkard, 1964; Werding, 1969; Prevot and Bartoli, 1978). At the same time, several cercariae whose descriptions are present in the literature are considered as renicolid larvae (e.g. Martin and Gregory, 1951; Cable, 1956, 1963; James, 1968*a*, 1969; Martin, 1971; Sannia and James, 1977; Cannon, 1978, 1979; Hechinger, 2007, 2019; Martorelli *et al*., 2008; Flores *et al*., 2019). There are among them cercariae with contrasting morphotypes: from typical xiphidiocercariae (small styleted cercariae with a simple tail) to large non-styleted larvae of the Rhodometopa group with tail fins (Wright, 1953, 1956; Odening, 1962; Cable, 1963; Stunkard, 1971; Prevot and Bartoli, 1978). Such a high diversity of cercarial morphotypes within a small family is unusual for trematodes (Galaktionov and Dobrovolskij, 2003). This matter apparently requires clarification, all the more so, as the results of molecular studies are ambiguous: some molecular data confirm that the larvae of the Rhodometopa group belong to renicolids (Matos *et al*., 2019), while other data indicate the opposite (Heneberg *et al*., 2016).

Despite the contrasting differences in the morphotype, species identification of cercariae is problematic because they are morphologically very similar in closely related species. This is the case, in particular, of renicolid intramolluscan stages from intertidal snails *Littorina* spp. in the North Atlantic (NA). Stunkard and Shaw (1931) and Stunkard (1932) described cercariae *Cercaria parvicaudata* Stunkard and Shaw, 1931 and *Cercaria roscovita* Stunkard, 1932 from these molluscs, but they are extremely difficult to differentiate (Stunkard, 1950; Galaktionov and Skírnisson, 2000). After the life cycle of, presumably, *C. roscovita* was elucidated and the species was named *Renicola roscovitus* (Stunkard, 1932) Werding, 1969, it has been generally assumed that this is the dominant renicolid species using periwinkles as the first intermediate hosts in NA (Lauckner, 1980, 1983). In a study of cercariae from intertidal molluscs in Iceland, Galaktionov and Skírnisson (2000) recorded only larvae corresponding to *C. parvicaudata* described by Stunkard and Shaw (1931) and Stunkard (1950). No cercariae matching the description of *C. roscovita* have been found during long-term studies of the fauna of digenean intramolluscan stages associated with *Littorina* spp. at the coasts of NA and the North Pacific (NP) (K. V. Galaktionov, personal observation). All these observations indicate that the question of the species composition of renicolids in NA and NP should be revisited.

The aim of this study was to ascertain the species composition of renicolids using periwinkles as the first intermediate hosts and to determine their transmission routes into NA and NP. We used an integrative approach, combining the analysis of morphological and molecular data, which has been shown to be the most effective in addressing taxonomy, phylogeny and elucidation of digenean life cycles (Blasco-Costa and Poulin, 2017). Relatively few studies on renicolids have employed this approach (Skírnisson *et al*., 2002–2003–2003; Hechinger and Miura, 2014; O'Dwyer *et al*., 2014, 2015; Patitucci *et al*., 2015; Heneberg *et al*., 2016; Flores *et al*., 2019; Matos *et al*., 2019), and our study is an addition to their number. In the course of our research on trematodes from the nearshore areas of NA and NP seas, we have collected and analysed extensive material on both intramolluscan stages and adults of renicolids from coastal birds, including gulls. Based on this material, we ascertained the species composition of renicolids from gulls in NA and NP and outlined the ways towards the elucidation of some aspects of their classification, evolution and ways of host colonization and geographical expansion. In addition, we confirmed that the larvae of Rhodometopa group belonged to renicolids and suggested how the cercariae of this type could have originated during the evolution of the taxon.

## Material and methods

### Material collection and treatment

The material presented in this study was collected from definitive and intermediate hosts (birds and molluscs) in 2002–2021 on the Atlantic coasts of Europe and North America and Pacific coast of North Asia (Table 1). Gastropod molluscs *Littorina saxatilis* (Olivi, 1792) and *Littorina obtusata* (Linnaeus, 1758) were collected in the intertidal zone of the White Sea, Barents Sea (Eastern Murman and Finmark) and Iceland, *Littorina sitkana* Philippi, 1846, in the Sea of Okhotsk (Magadan region) and *Littorina littorea* (Linnaeus, 1758), in the White Sea and the North Sea (Texel, the Netherlands) (Table 1). We also included in the molecular analysis *C. parvicaudata* isolates from *L. littorea* collected at the coasts of North East Atlantic (NEA) and North West Atlantic (NWA) during the study by Blakeslee and Byers (2008), with the sequence data reported in Blakeslee and Fowler (2012), in the summer months between 2002 and 2005 (Table 1). Herring gull *Larus argentatus* Pontoppidan, 1763 and slaty-backed gull *Larus schistisagus* Stejneger, 1884 were obtained by shooting in accordance with local regulations in South-West Iceland (Reykjavik region) and the Sea of Okhotsk (Magadan region), correspondingly.

**Table 1.** List of samples used in this study and corresponding GenBank accession numbers

Sample ID	Host species	Place	Region	Coordinates	GenBank accession numbers		
					28S D1–D3 fragment	cox1	ITS2
1siOP	*L. sitkana*	Veselaya Bay	Sea of Okhotsk, Russia	59°29.701′ N 150°55.176′ E	ON650718	ON652703	–
4IMR	*L. argentatus*	Akrakot	NE Atlantic, SW Iceland	64°18.270′ N 22°2.349′ W	–	ON652704	–
7saxIP	*L. saxatilis*	Akrakot	NE Atlantic, SW Iceland	64°18.315′ N 22°2.319′ W	ON650719	ON652705	ON667890
8OmR	*L. schistisagus*	Cape Njuklya	Sea of Okhotsk, Russia	59°29.700′ N 151°4.282′ E	ON650720	ON652706	–
10nIR	*N. lapillus*	Grotta	NE Atlantic, SW Iceland	64°9.606′ N 22°1.018′ W	ON650721	ON652707	ON667891
13saxWSP	*L. saxatilis*	Kem-Ludy archipelago	White Sea, Russia	66°25.107′ N 33°48.530′ E	–	ON652708	ON667892
14obtWSP	*L. obtusata*	Korga Islet	White Sea, Russia	66°18.061′ N 33°27.473′ E	–	ON652709	ON667893
26saxBP	*L. saxatilis*	Yarnyshnaya Bay	Barents Sea, Russia	69°5.232′ N 36°3.303′ E	ON650722	ON652710	ON667894
27litHR	*L. littorea*	Texel	Wadden Sea, Netherlands	53°0.115′ N 4°47.359′ E	ON650723	ON652711	ON667895
31litWSR	*L. littorea*	Cape Krasnyi	White Sea, Russia	66°24.664′ N, 33°42.911′ E	–	ON652712	–
32saxIC	*L. saxatilis*	Grindavik	NE Atlantic, SW Iceland	63°50.494′ N 22°25.194′ W	ON650724	ON652713	–
41saxBP	*L. saxatilis*	Yarnyshnaya Bay	Barents Sea, Russia	69°5.283′ N 36°3.374′ E	–	ON652714	–
42saxBP	*L. saxatilis*	Yarnyshnaya Bay	Barents Sea, Russia	69°5.169′ N 36°3.142′ E	–	ON652715	–
43saxBP	*L. saxatilis*	Yarnyshnaya Bay	Barents Sea, Russia,	69°5.161′ N 36°3.120′ E	–	ON652716	–
57obtBP	*L. obtusata*	Dalnezelenetskaya Bay	Barents Sea, Russia,	69°07.414′ N 36°5.892′ E	ON650725	ON652717	–
58siOP	*L. sitkana*	Veselaya Bay	Sea of Okhotsk, Russia	59°29.701′ N 150°55.176′ E	ON650726	ON652718	–
EUCPESBJE1	*L. littorea*	Esbjerg	Wadden sea, Denmark	55°28.859″ N 08°24.625′ E	–	ON652636	–
EUCPESBJE2	*L. littorea*	Esbjerg	Wadden sea, Denmark	55°28.859′ N 08°24.625′ E	–	ON652637	–
EUCPDUBIR1	*L. littorea*	Dublin	Irish sea, Ireland	53°19.10′ N 06°06.58′ W	–	ON652638	–
EUCPDUBIR2	*L. littorea*	Dublin	Irish sea, Ireland	53°19.10′ N 06°06.58′ W	–	ON652639	–
EUCPDUBIR3	*L. littorea*	Dublin	Irish sea, Ireland	53°19.10′ N 06°06.58′ W	–	ON652640	–
EUCPDUBIR4	*L. littorea*	Dublin	Irish sea, Ireland	53°19.10′ N 06°06.58′ W	–	ON652641	–
EUCPMINDI1	*L. littorea*	Mindin	NE Atlantic, France	47°16.112′ N 02°10.262′ W	–	ON652642	–
EUCPMINDI2	*L. littorea*	Mindin	NE Atlantic, France	47°16.112′ N 02°10.262′ W	–	ON652643	–
EUCPMINDI3	*L. littorea*	Mindin	NE Atlantic, France	47°16.112′ N 02°10.262′ W	–	ON652644	–
EUCPMINDI4	*L. littorea*	Mindin	NE Atlantic, France	47°16.112′ N 02°10.262′ W	–	ON652645	–
EUCPMINDI5	*L. littorea*	Mindin	NE Atlantic, France	47°16.112′ N 02°10.262′ W	–	ON652646	–
EUCPMOSSN1	*L. littorea*	Moss	Oslofjord, Norway	59°25.861′ N 10°39.148′ E	–	ON652647	–
EUCPMOSSN2	*L. littorea*	Moss	Oslofjord, Norway	59°25.861′ N 10°39.148′ E	–	ON652648	–
EUCPMOSSN3	*L. littorea*	Moss	Oslofjord, Norway	59°25.861′ N 10°39.148′ E	–	ON652649	–
EUCPMOSSN4	*L. littorea*	Moss	Oslofjord, Norway	59°25.861′ N 10°39.148′ E	–	ON652650	–
EUCPMOSSN5	*L. littorea*	Moss	Oslofjord, Norway	59°25.861′ N 10°39.148′ E	–	ON652651	–
EUCPMOSSN6	*L. littorea*	Moss	Oslofjord, Norway	59°25.861′ N 10°39.148′ E	–	ON652652	–
EUCPMOSSN7	*L. littorea*	Moss	Oslofjord, Norway	59°25.861′ N 10°39.148′ E	–	ON652653	–
EUCPMOSSN8	*L. littorea*	Moss	Oslofjord, Norway	59°25.861′ N 10°39.148′ E	–	ON652654	–
EUCPOSTEN1	*L. littorea*	Ostende	Nothern sea, Belgium	51°13.593′ N 02°56.596′ E	–	ON652655	–
EUCPOSTEN2	*L. littorea*	Ostende	Nothern sea, Belgium	51°13.593′ N 02°56.596′ E	–	ON652656	–
EUCPOSTEN3	*L. littorea*	Ostende	Nothern sea, Belgium	51°13.593′ N 02°56.596′ E	–	ON652657	–
EUCPTJARN1	*L. littorea*	Tjarno	Skagerrak, Sweden	58°53.107′ N 11°07.117′ E	–	ON652658	–
EUCPTJARN2	*L. littorea*	Tjarno	Skagerrak, Sweden	58°53.107′ N 11°07.117′ E	–	ON652659	–
EUCPTJARN3	*L. littorea*	Tjarno	Skagerrak, Sweden	58°53.107′ N 11°07.117′ E	–	ON652660	–
EUCPTJARN4	*L. littorea*	Tjarno	Skagerrak, Sweden	58°53.107′ N 11°07.117′ E	–	ON652661	–
EUCPTROUV1	*L. littorea*	Trouville	English Channel, France	49°21.851′ N 00°04.871′ E	–	ON652662	–
EUCPTROUV2	*L. littorea*	Trouville	English Channel, France	49°21.851′ N 00°04.871′ E	–	ON652663	–
EUCPTROUV3	*L. littorea*	Trouville	English Channel, France	49°21.851′ N 00°04.871′ E	–	ON652664	–
EUCPUBDJH1	*L. littorea*	Udbyhoj	Kattegat, Denmark	56°36.565′ N 10°17.986′ E	–	ON652665	–
EUCPUBDJH2	*L. littorea*	Udbyhoj	Kattegat, Denmark	56°36.565′ N 10°17.986′ E	–	ON652666	–
EUCPUBDJH3	*L. littorea*	Udbyhoj	Kattegat, Denmark	56°36.565′ N 10°17.986′ E	–	ON652667	–
EUCPUBDJH4	*L. littorea*	Udbyhoj	Kattegat, Denmark	56°36.565′ N 10°17.986′ E	–	ON652668	–
EUCPUBDJH5	*L. littorea*	Udbyhoj	Kattegat, Denmark	56°36.565′ N 10°17.986′ E	–	ON652669	–
EUCPVARBE1	*L. littorea*	Varberg	Kattegat, Sweden	56°36.565′ N 10°17.986′ E	–	ON652670	–
EUCPVARBE2	*L. littorea*	Varberg	Kattegat, Sweden	56°36.565′ N 10°17.986′ E	–	ON652671	–
NACPBOOTH2	*L. littorea*	Boothbay	NW Atlantic, USA	43°50.55′ N 69°37.55′ W	–	ON652672	–
NACPBOOTH5	*L. littorea*	Boothbay	NW Atlantic, USA	43°50.55′ N 69°37.55′ W	–	ON652673	–
NACPBOOTH6	*L. littorea*	Boothbay	NW Atlantic, USA	43°50.55′ N 69°37.55′ W	–	ON652674	–
NACPCPMAY1	*L. littorea*	Cape May	NW Atlantic, USA	38°57.349′ N 74°52.568′ W	–	ON652675	–
NACPCPMAY2	*L. littorea*	Cape May	NW Atlantic, USA	38°57.349′ N 74°52.568′ W	–	ON652676	–
NACPCPMAY3	*L. littorea*	Cape May	NW Atlantic, USA	38°57.349′ N 74°52.568′ W	–	ON652677	–
NACPCPMAY4	*L. littorea*	Cape May	NW Atlantic, USA	38°57.349′ N 74°52.568′ W	–	ON652678	–
NACPHALIF1	*L. littorea*	Halifax	NW Atlantic, USA	44°37.479′ N 63°33.850′ W	–	ON652679	–
NACPHALIF2	*L. littorea*	Halifax	NW Atlantic, USA	44°37.479′ N 63°33.850′ W	–	ON652680	–
NACPHALIF3	*L. littorea*	Halifax	NW Atlantic, USA	44°37.479′ N 63°33.850′ W	–	ON652681	–
NACPMONTA1	*L. littorea*	Montauk	NW Atlantic, USA	41°04.309′ N 71°51.501′ W	–	ON652682	–
NACPMONTA2	*L. littorea*	Montauk	NW Atlantic, USA	41°04.309′ N 71°51.501′ W	–	ON652683	–
NACPMONTA3	*L. littorea*	Montauk	NW Atlantic, USA	41°04.309′ N 71°51.501′ W	–	ON652684	–
NACPMONTA4	*L. littorea*	Montauk	NW Atlantic, USA	41°04.309′ N 71°51.501′ W	–	ON652685	–
NACPMONTA5	*L. littorea*	Montauk	NW Atlantic, USA	41°04.309′ N 71°51.501′ W	–	ON652686	–
NACPODIOR1	*L. littorea*	Odiorne, Rye	Gulf of Maine, USA	43°00.215′ N 70°44.986′ W	–	ON652687	–
NACPPTJRI1	*L. littorea*	Point Judith	NW Atlantic, USA	41°21.767′ N 71°28.828′ W	–	ON652688	–
NACPPTJRI2	*L. littorea*	Point Judith	NW Atlantic, USA	41°21.767′ N 71°28.828′ W	–	ON652689	–
NACPPTJRI3	*L. littorea*	Point Judith	NW Atlantic, USA	41°21.767′ N 71°28.828′ W	–	ON652690	–
NACPSPOND1	*L. littorea*	Vineyard Haven	NW Atlantic, USA	41°27.520′ N 70°35.164′ W	–	ON652691	–
NACPSPOND2	*L. littorea*	Vineyard Haven	NW Atlantic, USA	41°27.520′ N 70°35.164′ W	–	ON652692	–
NACPSPOND3	*L. littorea*	Vineyard Haven	NW Atlantic, USA	41°27.520′ N 70°35.164′ W	–	ON652693	–
NACPSPOND4	*L. littorea*	Vineyard Haven	NW Atlantic, USA	41°27.520′ N 70°35.164′ W	–	ON652694	–
NACPWELLS1	*L. littorea*	Wells	Gulf of Maine, USA	43°20.067′ N 70°32.554′ W	–	ON652695	–
NACPWELLS2	*L. littorea*	Wells	Gulf of Maine, USA	43°20.067′ N 70°32.554′ W	–	ON652696	–
NACPWELLS3	*L. littorea*	Wells	Gulf of Maine, USA	43°20.067′ N 70°32.554′ W	–	ON652697	–
NACPYORKM1	*L. littorea*	York	Gulf of Maine, USA	43°20.067′ N 70°32.554′ W	–	ON652698	–
NACPYORKM2	*L. littorea*	York	Gulf of Maine, USA	43°20.067′ N 70°32.554′ W	–	ON652699	–
NACPYORKM3	*L. littorea*	York	Gulf of Maine, USA	43°20.067′ N 70°32.554′ W	–	ON652700	–
11 Ersh	*L. littorea*	Woods Hole, MA	Cape Cod, USA	41°31.502′ N; 70°40.403′ W	–	ON652701	–
12 Ersh	*L. littorea*	Woods Hole, MA	Cape Cod, USA	41°31.502′ N; 70°40.403′ W	–	ON652702	–

*L. littorea*, *Littorina littorea*; *L. saxatilis*, *Littorina saxatilis*; *L. argentatus*, *Larus argentatus*; *L. schistisagus*, *Larus schistisagus*; *N. lapillus*, *Nucella lapillus*

The molluscs were dissected under a stereomicroscope to identify those infected with renicolid intramolluscan stages. Some snails were placed in plastic jars filled with seawater (1 snail per jar) and exposed to sunlight or direct artificial light for 1 h. The jars were examined under a stereomicroscope and the individuals that had shed cercariae of *Renicola* spp. were selected. These snails, kept in the refrigerator under 4°C, were used as a source of cercariae, which were obtained when required following the same procedure as in case of freshly collected snails.

The species of renicolid intramolluscan stages was identified on the basis of the original descriptions by Stunkard and Shaw (1931) and Stunkard (1932, 1950). Live sporocysts and cercariae were observed, measured and photographed using Olympus CH40 compound microscope equipped with an Olympus XC-30 digital camera at the ‘Kartesh’ White Sea Biological Station of the Zoological Institute of the Russian Academy of Science (ZIN RAN); Leica compound microscope in the Institute of Pathology (Keldur, Iceland) and Leitz Dialux 20B compound microscope in the Institute of Biological Problems of the North (Magadan, Russia). Only newly shed cercariae were used for morphometric studies and scanning electron microscopy (SEM). Cercariae to be measured were fixed by heating in a drop of seawater on the object slide (until the water started to evaporate), and then gently pressed with a coverslip. Sporocysts and encysted metacercariae were measured *in vivo*. For SEM, we used cercariae *C. parvicaudata* newly shed from the White Sea *L. littorea*. Cercariae fixation procedure and treatment before SEM examination were done as described in Galaktionov *et al*. (2021). The treated cercariae were viewed under a FEI Quanta 250 scanning electron microscope in ‘Taxon’ Research Resource Center (http://www.ckp-rf.ru/ckp/3038/) of ZIN RAS. For molecular studies, we used renicolid intramolluscan stages whose species had been tentatively identified based on morphological criteria. This material was fixed in 95% ethanol.

Gulls were dissected and the renicolid individuals were extracted from the kidney. These adults were fixed in 70% ethanol under a slight pressure of a coverslip. Samples of adults were stored in 70 and 95% ethanol for further morphological and molecular analysis, correspondingly. Carmine-stained whole mounts were used for morphological studies, to make drawings and photographs using Leica DM2500 compound microscope with camera lucida and ToupCam UCMOS14000 digital camera. All measurements presented in the paper are in micrometres, with the mean in parentheses. Drawings were made with the aid of camera lucida.

### DNA extraction, amplification and sequencing

We determined the sequences of 28S ribosomal RNA (rRNA) and *cox1* mitochondrial genes for rediae and cercariae of *Renicola* spp. from infected periwinkles and birds (Table 1). Genomic DNA was extracted with cetrimonium bromide (CTAB) detergent according to the published protocol with modifications (Winnepenninckx *et al*., 1993) from ethanol-fixed isolates. Fixed specimens were rinsed in 1× phosphate-buffered saline for 15 min before extraction. The D1–D3 fragment of 28S rRNA gene was amplified with primers ZX-1 (5′-ACCCGCTGAATTTAAGCATAT-3′) (Palm *et al*., 2009) and 1500R (5′-GCTATCCTGAGGGA AACTTCG-3′) (Olson *et al*., 2003) according to the following temperature profile: initial DNA denaturation at 95°C for 5 min, then 30 cycles (95°C for 1 min; 55°C for 30 s; 72°C for 1 min) and a final elongation step at 72°C for 5 min. The *cox1* gene fragments were amplified with primers JB3 (5′-TTTTTTGGGCATCCTGAGGTTTAT-3′) и JB4-5 (5′-TAAAGAAAGAACATAATGAAAATG-3′) (Bowles *et al*., 1992) with the following conditions: initial DNA denaturation at 95°C for 5 min, then 30 cycles (95°C for 1 min; 53°C for 30 s; 72°C for 45 s) and a final elongation step at 72°C for 5 min. PCR reactions were run on the Mastercycler personal 5332 (Eppendorf, USA) thermal cycler. ITS2 fragment was amplified with NC13(ITS2)/F(5′-ATC GAT GAA GAA CGC AGC-3′) и Dd28SR1(5′-ACA AAC AAC CCG ACT CCA AG-3′) primers according to Heneberg *et al*. (2016). PCR products were purified following a modified protocol (Dyachenko *et al*., 2008; Galaktionov *et al*., 2021). DNA sequencing was performed at the Development of Molecular and Cellular Technologies Resource Centre at St. Petersburg State University and the University of New Hampshire (Durham, New Hampshire, USA). Two *cox1* gene sequences of samples from NWA *L. littorea* recognized as *R. roscovita* were kindly provided by Natalia Ershova (University of Chicago). All the sequences obtained in this study were deposited in GenBank (Table 1).

### Alignments and phylogenetic analyses

We performed alignment, trimming and basic analyses in Geneious 7.1.4 http://www.geneious.com (Kearse *et al*., 2012) of the newly generated sequences together with 28S rRNA gene and *cox1* partial sequences retrieved from GenBank for other *Renicola* spp. Genetic divergences among taxa were calculated as uncorrected *p*-distances for each gene region using MEGA v. X (Tamura *et al*., 2013). Phylogenetic relationships were reconstructed using Bayesian inference (BI) on MrBayes v. 3.2.6 (Ronquist *et al*., 2012) and maximum likelihood (ML) on MEGA X (Kumar *et al*., 2018). The most suitable evolutionary models were determined by the corrected Akaike information criterion in the PartitionFinder program (https://github.com/brettc/partitionfinder). The Hasegawa–Kishino–Yano model with estimates of gamma-distributed among-site rate variation (HKY + G) was chosen as best fitted for *cox1* gene. Kimura 2-parameter model with estimates of gamma-distributed among-site rate variation was chosen for fragments of 28S rRNA genes. Genetic divergences among taxa were calculated as uncorrected *p*-distances for each gene region using MEGA X (Kumar *et al*., 2018). Mismatch distribution and Tajima's *D* neutrality test were calculated in DNASP 6 program (Rozas *et al*., 2017). We also performed the species partitioning with clustering algorithm implemented in ASAP tool (Puillandre *et al*., 2020). Haplotype network was reconstructed with PopArt tool (Leigh and Bryant, 2015).

## Results

Molecular results showed that renicolid intramolluscan stages from *L. littorea* and *L. sitkana* and most isolates from *L. obtusata* and *L. saxatilis*, identified as *C. parvicaudata* based on morphological criteria, belonged to one and the same species. Their sequences also matched that of the adult from the Icelandic herring gull, which made it possible to complete the life cycle of this species. We named it *Renicola parvicaudatus* (Stunkard and Shaw, 1931) nov. comb. (see Molecular results and Remarks for details). Among the isolates from *L. obtusata* and *L. saxatilis*, initially identified as *C. parvicaudata*, the analysis of molecular markers made it possible to differentiate intramolluscan stages of the cryptic species, which we named *Cercaria littorinae saxatilis* VIII larva nov. In slaty-backed gulls of the Sea of Okhotsk, besides *R. parvicaudatus*, we found the adults of one more *Renicola* species, which we identified as *Renicola keimahuri* Yamaguti, 1939.

*Description*

Family Renicolidae Dollfus, 1939

*Renicola parvicaudatus* (Stunkard and Shaw, 1931) nov. comb.

[syn. *C. parvicaudata* Stunkard and Shaw, 1931, *R. roscovitus* (Stunkard, 1932) Werding, 1969; sexual adults of *Renicola thaidus* Stunkard, 1964].

ZooBank LSID: urn:lsid:zoobank.org:pub:86EDD019-DF69-487C-A6C9-DF790F43966D

Type host (definitive): herring gull *L. argentatus* Pontoppidan, 1763, slaty-backed gull *L. schistisagus* Stejneger, 1884 (Laridae).

Site in definitive host: kidney.

Type-locality: South-West Iceland.

Other localities (in definitive host): Nagaeva Bay, Sea of Okhotsk.

Type material: 11 syntypes (on slides 3732-1, 3732-2, 3733-1, 3733-2, 3734-1 and 3734-2), deposited in the Collection of Helminths, section Trematoda, of the Zoological Institute of the Russian Academy of Sciences, St. Petersburg, Russia. This material represents paragenophores.

First intermediate host: *L. littorea* (Linnaeus, 1758), *L. saxatilis* (Olivi, 1792), *L. obtusata* (Linnaeus, 1758) and *L. sitkana* Philippi, 1846 (Caenogastropoda: Littorinimorpha: Littorinidae) (natural).

Site in first intermediate host: gonad.

Localities (in first intermediate host): NEA, NWA, NP.

Second intermediate host: *Mytilus edulis* (Linnaeus, 1758), *Cerastoderma edule* (Linnaeus, 1758), *Argopecten irradians irradians* (Lamarck, 1819), occasionally *L. littorea*, *L. saxatilis* and *L. obtusata*.

Representative DNA sequences: 28S rDNA (ON650718, ON650721, ON650723, ON650724, ON650726), *cox1* (ON652703, ON652704, ON652707–ON652709, ON652711–ON652713, ON652718, ON652636–ON652702) and ITS2 rDNA (ON667891–ON667893, ON667895) (according to Table 1).

**Fig. 1.**
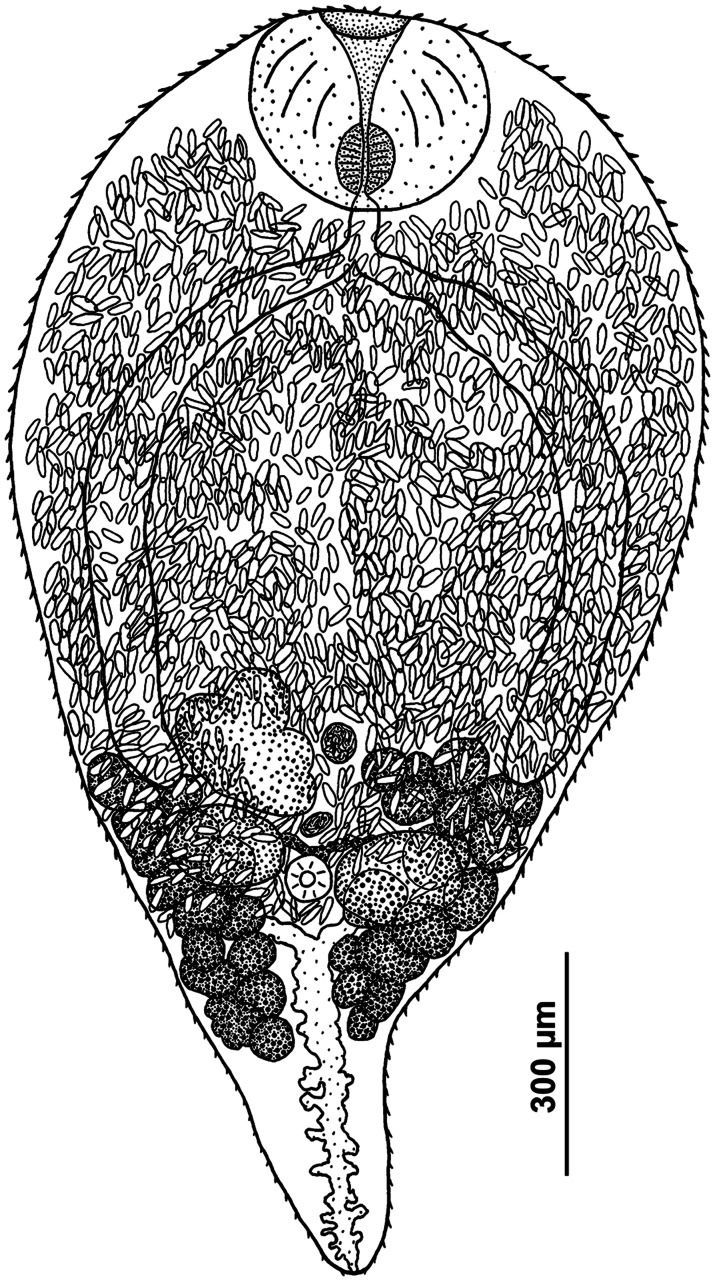
Sexual adult of *R. parvicaudatus* (ventral view).

**Fig. 2.**
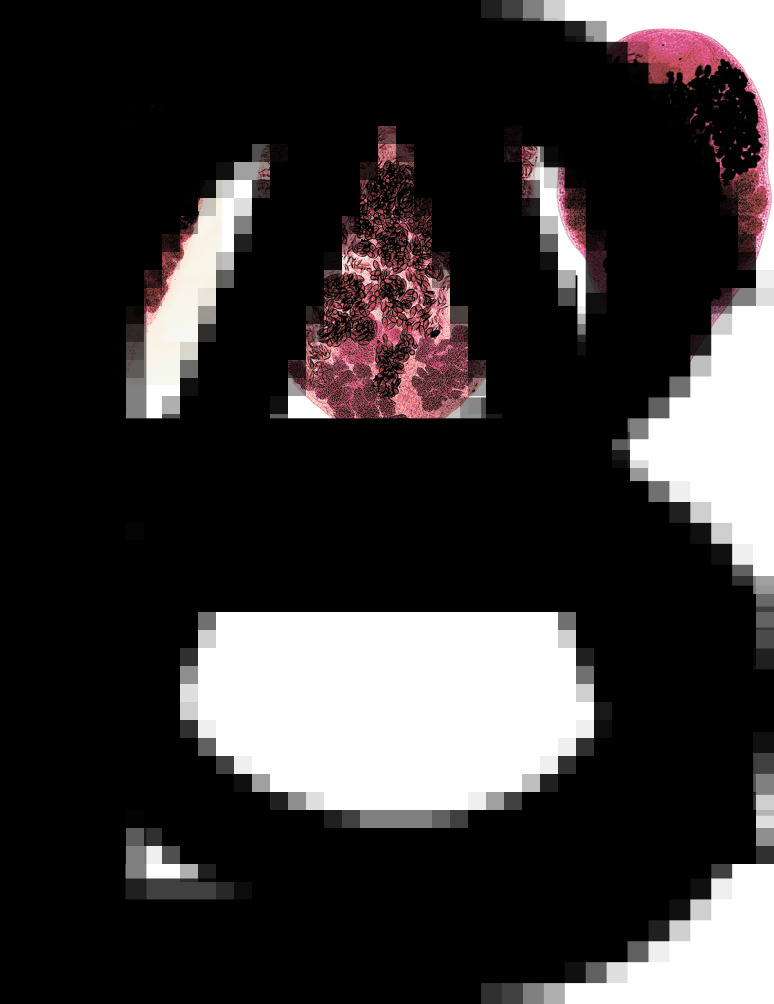
Representative microphotographs of sexual adults of *Renicola* spp. analysed in this study (ventral view): *R. parvicaudatus* from the Icelandic *Larus argentatus* (A); *R. parvicaudatus* (specimen heavily pressed by cover glass) from *L. schistisagus* of the Sea of Okhotsk (B); *R. keimahuri* from *L. schistisagus* of the Sea of Okhotsk (C).

*Sexual adults* (Table 2, Figs 1 and 2)

**Table 2.** Morphometric parameters of adults of *Renicola* spp. parasitizing gulls

	*R. parvicaudatus* Our data (from *Larus argentatus*, Iceland, *N* = 5)	*R. parvicaudatus* Our data (from *Larus schistisagus*, Sea of Okhotsk, *N* = 3)	*R. roscovitus* After Werding (1969) (from *Larus argentatus*)	*R. murmanicus* After Belopol'skaya (1952) (from *Larus argentatus*)	*R. thaidus* After Stunkard (1964) (from *Larus argentatus*)	*R. keimahuri* Our data (from *Larus schistisagus*, Sea of Okhotsk, *N* = 18)	*R. keimahuri* After Yamaguti (1939) (from *Cepphus carbo*)	*R. sternae* After Heneberg *et al*. (2016) (from *Sterna hirundo*)	*R. lari* After Prevot and Bartoli (1978) (from *Larus argentatus*)
Body length	850–1680 (1173 ± 156)	1005–1550 (1321 ± 163)	960–1340	528–1143	700–1160	593–1218 (923 ± 39)	1150–2100	571–1629 (1103 ± 243)	1225–1945 (1448)
Body width	429–1062 (705 ± 107)	558–975 (793 ± 123)	575–805	530–580	400–600	363–868 (637 ± 34)	470–1000	514–1057 (785 ± 158)	560–1039 (823)
Oral sucker length	100–218 (165 ± 24)	268–346 (312 ± 40)	210–240	159–185*	260–300*	130–367 (198 ± 13)	150–200*	145–285 (198 ± 43)	158–227 (193)
Oral sucker width	111–323 (218 ± 40)	292–387 (338 ± 27)	250–295			105–360 (239 ± 20)		174–368 (242 ± 67)	195–270 (232)
Pharynx length	50–83 (70 ± 5)	68–83 (76 ± 4)	42–67*	29–31	52–60*	64–107 (80 ± 3)	40–60*	48–87 (67 ± 11)	65–86 (76)
Pharynx width	50–73 (63 ± 4)	60–73 (65 ± 4)		35–36		60–120 (79 ± 4)		36–87 (65 ± 11)	54–80 (65)
Oesophagus length	–	63–130 (95 ± 19)				82–130 (106 ± 24)			
Ventral sucker length	32–75 (49 ± 8)	–		34–41*	20*	82–109 (92 ± 3)	60–70*	84–108 (96 ± 6)	76–101 (85)
Ventral sucker width	30–75 (45 ± 8)	–				74–104 (91 ± 3)		84–110 (96 ± 6)	72–102 (87)
Left testes length	79–136 (108 ± 14)	83–107 (97 ± 7)	35–50*	49–50	60–90*	58–150 (94 ± 9)	110–120	80–116 (97 ± 14)	130–220 (165)
Left testes width	53–126 (81 ± 17)	60–69 (64 ± 3)		47–49		36–82 (51 ± 4)	90	58–74 (66 ± 11)	32–117 (75)
Right testes length	72–170 (125 ± 23)	81–100 (90 ± 5)	35–50*	49–50	60–90*	50–148 (95 ± 8)	110–120	80–116 (95 ± 13)	100–217 (163)
Right testes width	50–134 (91 ± 23)	45–69 (59 ± 7)		47–49		26–78 (53 ± 5)	90	58–74 (66 ± 11)	48–135 (177)
Seminal vesicle length	43–77 (56 ± 11)	50–71 (59 ± 6)				22–37 (31 ± 5)	35*		
Seminal vesicle width	27–60 (40 ± 10)	24–50 (35 ± 8)				26–35 (31 ± 3)			
Ovary length	118–214 (162 ± 23)	125–180 (149 ± 16)	150–210	139	160–240	91–280 (172 ± 13)	260–290	87–261 (199 ± 55)	256–435 (357)
Ovary width	103–180 (137 ± 17)	130–214 (158 ± 28)	115–180	90	120–160	30–152 (107 ± 10)	110–130	87–145 (112 ± 14)	80–238 (146)
Seminal receptacle length	53					36–50 (43 ± 7)	38*		
Seminal receptacle width	44	–				33–35 (34 ± 1)			
Left vitellaria length	142–548 (356 ± 82)	324–418 (358 ± 30)				165–376 (264 ± 18)	340 (max–500)	261–506 (352 ± 63)	205–540 (358)
Right vitellaria length	212–394 (299 ± 33)	233–447 (345 ± 62)				177–331 (246 ± 11)	220 (max–500)	261–506 (352 ± 63)	217–590 (391)
Egg length (EL)	38–48 (43 ± 0.8)	41–49 (46 ± 0.6)	42–47 [49–50]	38–42	36–38 [42]	33–40 (37 ± 0.4)	39–45	32–36 (35 ± 2)	28–36 (33.3)
Egg width (EW)	13–19 (16 ± 0.5)	16–21 (19 ± 0.3)	20–22 [20–23]	18–19	16–17 [20]	16–23 (19 ± 0.4)	20–22	17–24 (22 ± 3)	15–21 (18)
EL/EW	2.2–3.5 (2.7 ± 0.1)	2.0–3.1 (2.5 ± 0.1)				1.6–2.6 (2.0 ± 0.05)		(1.6)	

Host species is indicated in brackets in the column heads; N, number of measured individuals; * diameter of organs; measurements of live worms are given in square brackets.

The description is based on morphologically identical adults from herring gull obtained in South-West Iceland. One of the adult worms matched intramolluscan stages of *C. parvicaudata* in the marker DNA sequences.

Body ovoid, rounded anteriorly and attenuated posteriorly. Size of worms varying greatly depending on number of eggs in uterus. Oral sucker subterminal to terminal, transversely elongated-oval. Ventral sucker 3–5 times smaller than oral sucker, in posterior third of body. Ventral sucker poorly discernible in large worms with numerous eggs. Prepharynx absent; pharynx small, often deeply embedded in wall of oral sucker. Oesophagus short, caeca 2, extending into posterior third of body. Testes oval, lying laterally of the ventral sucker, more or less opposite to each other. Left testis somewhat larger than right testis. Seminal vesicle lying anteriorly of ventral sucker approximately at level of middle to anterior part of ovary, median or lightly dextral of body midline. Ovary dextral (rarely sinistral), pretesticular, larger than testes, variously lobed. Seminal receptacle median or lightly dextral, just anterior to ventral sucker. Uterus strongly developed, occupying most of body. Eggs numerous, elongated (length about 3 times greater than width), operculate, with thin eggshell. Vitellarium follicular; follicles in 2 lateral fields in posterior third of body extended from the base of attenuated posterior part of body to level of middle or anterior border of ovary; consisting of 10–18 large follicles on ovarian side of body and 13–18 on opposite side; follicles most often fusing together. Excretory bladder Y-shaped, with distinct lateral diverticula; bifurcates just posterior to the ventral sucker, arms extending into forebody up to level of oral sucker.

*Intramolluscan stages*

The description is based on examination of intramolluscan stages from *L. littorea* collected in Texel (the Netherlands) and in the White Sea, from *L. saxatilis* and *L. obtusata* collected in Iceland (Reykjavik region) and in the White Sea, and from *L. sitkana* collected in the Sea of Okhotsk (Nagaeva Bay). Intramolluscan stages isolated from each snail were conspecific, as confirmed by the analysis of the molecular markers.

*Sporocyst* [measurements based on 30 live specimens]

**Fig. 3.**
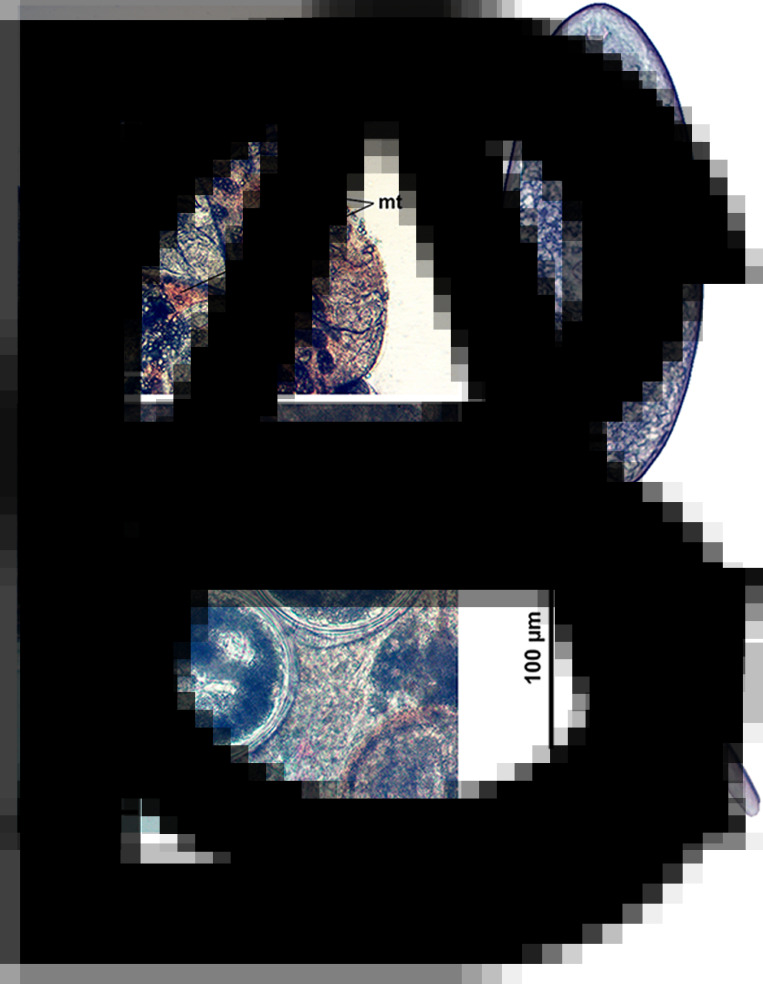
Microphotographs of the intramolluscan stages and cercaria of *R. parvicaudatus*: daughter sporocysts in the gonad of *Littorina littorea* (A); cercaria (B) and metacercariae encysted in the same molluscan host where daughter sporocysts develop (C). mt, Molluscan tissue; sp, daughter sporocysts.

Sporocyst (Fig. 3A) elongate oval, 437–876 × 213–444 (641 ± 25 × 345 ± 9), containing 1–12 (4) motile cercariae and numerous embryos. Sporocysts occupy the molluscan gonad tissue forming a tumour-like structure. The pseudo-tumour, milky white in case of early infection, becomes lemon-yellow or orange as cercariae mature in the sporocysts. The pigment responsible for the colour of the tumour is mostly concentrated in the surrounding host tissue, not in the sporocyst wall.

**Fig. 4.**
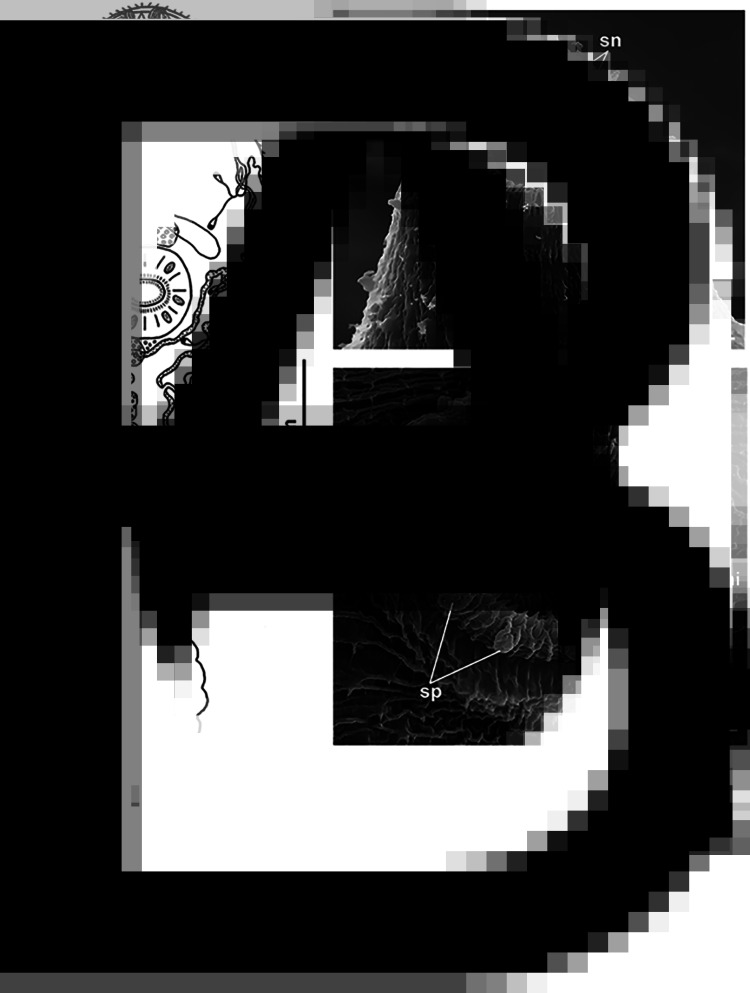
Cercaria of *R. parvicaudatus*: drawings of cercaria (ventral view) (A) and stylet (ventral view) (B); SEM microphotographs showing spines in oral (C) and ventral sucker (D). sn, Spines; sne, spines of the external row; sni, spines of the internal row; sp, uniciliated sensory papillae surrounded by wide convex tegumental collars.

*Cercaria* (Table 3, Figs 3B and 4)

**Table 3.** Morphometric parameters of cercariae of *R. parvicaudatus* and closely related species (‘Parvicaudata’ group)

	*R. parvicaudatus* Our data (*N* = 31)	*C. littorinae saxatilis* VIII Our data (*N* = 20)	*C. parvicaudata* After Stunkard (1950)	*C. roscovita* After Stunkard (1950)	*R. roscovitus* After Werding (1969)	*C. roscovita* After James (1969)	*Renicola* sp. NZ After O'Dwyer *et al*. (2014)	*Renicola* sp. 1 Aus After O'Dwyer *et al*. (2015)	*Renicola* sp. 2 Aus After O'Dwyer *et al*. (2015)
Body length	189–333 (262 ± 6.2)	218–338 (280 ± 6.8)	140–360	150–300	129–330 [240]	280–350	205–264 (240)	239–307 (268)	226–310 (263)
Body width	73–143 (101 ± 3.5)	88–120 (101 ± 2.3)	60–120	60–120	45–135 [89]	80–100	77–101 (86)	71–90 (82)	77–130 (107)
Tail length	155–197 (175 ± 2)	135–203 (181 ± 3.4)	60–300	80–300	44–240 [148]	50–300	150–207 (166)	168–222 (193)	124–189 (154)
Tail width	16–26 (21 ± 0.4)	16–21 (20 ± 0.6)	–	–	max. 33 [19]	–	16–24 (19)	15–21 (18)	16–19 (18)
Oral sucker length	45–60 (51 ± 1.2)	38–50 (44 ± 0.9)	35–60*	42–50*	36–39* [42]	35–50*	33–40 (37)	33–44 (36)	36–51 (43)
Oral sucker width	40–58 (46 ± 1.3)	33–45 (41 ± 0.8)	–	–	[33]	–	29–37 (33)	28–35 (32)	33–43 (37)
Pharynx length	15–25 (20 ± 0.7)	13–20 (15 ± 0.4)	12–14*	14–18*	14	10–18*	12	–	15
Pharynx width	15–23 (19 ± 0.5)	10–20 (15 ± 0.5)	–	–	14	–	12	–	9
Ventral sucker length	43–55 (48 ± 1)	35–45 (41 ± 0.9)	34–50*	34–50*	33–36 [35]	30–45*	30–36 (33)	30–41 (36)	29–47 (38)
Ventral sucker width	43–58 (48 ± 1.4)	33–53 (44 ± 1.1)	–	–	[38]	–	26–36 (32)	30–41 (34)	32–45 (37)
Stylet length	12–15 (13 ± 0.3)	12–17 (14 ± 0.4)	15	16–18	14	13–18	10–12	11	10–12 (12)
Stylet width 1	4–6 (5 ± 0.2)	–	–	–	–	–	1	2	3 (3)
Stylet width 2	3–4 (3 ± 0.1)	3–5 (4 ± 0.2)	3.2	2–3	3	–	1	2	

N, number of measured individuals; * diameter; measurements of live worms are given in square brackets. Stylet width 1 – width in the broad part of the spearhead; stylet width 2 – width of the handle.

Cercariae small, body oval, highly contractile, body length more than 1.5 times greater than tail length. Oral sucker ventro-subterminal, muscular, approximately the same size as ventral sucker. Oral sucker armed with a single row of 38–43 spines (Fig. 4C). Stylet spear-shaped with a weakly expressed light-refracting spearhead, dorsal to mouth opening (Fig. 4B). Ventral sucker equatorial, armed with 2 alternating rows of spines of 38–40 (Fig. 4D). Anteriorly to external row of spines, ventral sucker bears 6 characteristic short sensory papillae surrounded by wide convex tegumental collars (2 anterior and 4 posterior) (Fig. 4D).

Penetration gland cells numbering 6 pairs. Their nucleated bodies arranged symmetrically on either side of oesophagus approximately at level of its middle and posteriorly. Ducts skirting oral sucker dorsally and opening at each side with common bundle near external opening of stylet pocket. Anterior parts of ducts forming pronounced curve near anterior end of oral sucker (Fig. 4A). Contents of penetration gland cells finely granular, stained with neutral red.

Entire body of larva densely packed with tegumental cystogenous gland cells. Two types of these cells distinctly seen: cells with coarsely granular contents staining with neutral red and cells with granular unstaining contents. Cells of first type with distinct nuclei, nuclei in cells of second type indistinguishable. At final stages of larva formation gland cells apparently discharging some of contents into tegument, granular material being visible throughout body and not only in cells.

Prepharynx not pronounced, pharynx rounded, intestine short, bifurcating anteriorly of ventral sucker. Excretory bladder Y-shaped, its arms skirting the ventral sucker posteriorly. Main collecting tubes opening at either side into unpaired part of bladder close to its bifurcation. Excretory formula 2[(3 + 3 + 3) + (3 + 3 + 3)] = 36.

*Metacercaria* (Fig. 3C)

Metacercariae are enclosed in a spherical cyst 150–180 *μ*m in diameter; cyst wall is 10–20 *μ*m thick. The preferred second intermediate host is the mussel *M. edulis*. In mussels, the cysts with metacercariae are located in the hepatopancreas and, more rarely, in the tentacles at the mantle edge. The cercariae may also encyst in the same individuals of *Littorina* spp. that harbour daughter sporocysts. In this case they are located in the host tissues between the sporocysts. Encystment in periwinkles is more common during the cold season, after the arrest of cercarial emergence.

*Cercaria littorinae saxatilis* VIII larva nov. (Table 3)

First intermediate host: *L. saxatilis* and *L. obtusata* (Caenogastropoda: Littorinimorpha: Littorinidae) (natural).

Site in first intermediate host: gonad.

Localities (in first intermediate host): Dalniye Zelentsy, Barents Sea, Grindavik, South-West Iceland.

Representative DNA sequences: 28S rDNA (ON650719, ON650722, ON650725), *cox1* (ON652705, ON652710, ON652714–ON652717) and ITS2 rDNA (ON667894) (according to Table 1).

Etymology: the name of the intramolluscan stages continues the tradition of the classification of cercariae and parthenitae developing in molluscs *Littorina* spp., introduced by Lebour (1911) and continued by James (1968*b*, 1969), Sannia and James (1977) and Newell (1986).

The species was identified based on the analysis of molecular markers of intramolluscan stages from snails *L. saxatilis* collected in Iceland (Reykjavik region) and the Barents Sea (coast of the Kola Peninsula) (see molecular results). Daughter sporocysts and cercariae of *C. littorina saxatilis* VIII are morphologically and morphometrically identical to the intramolluscan stages of *R. parvicaudatus* described above (Table 3).

**Fig. 5.**
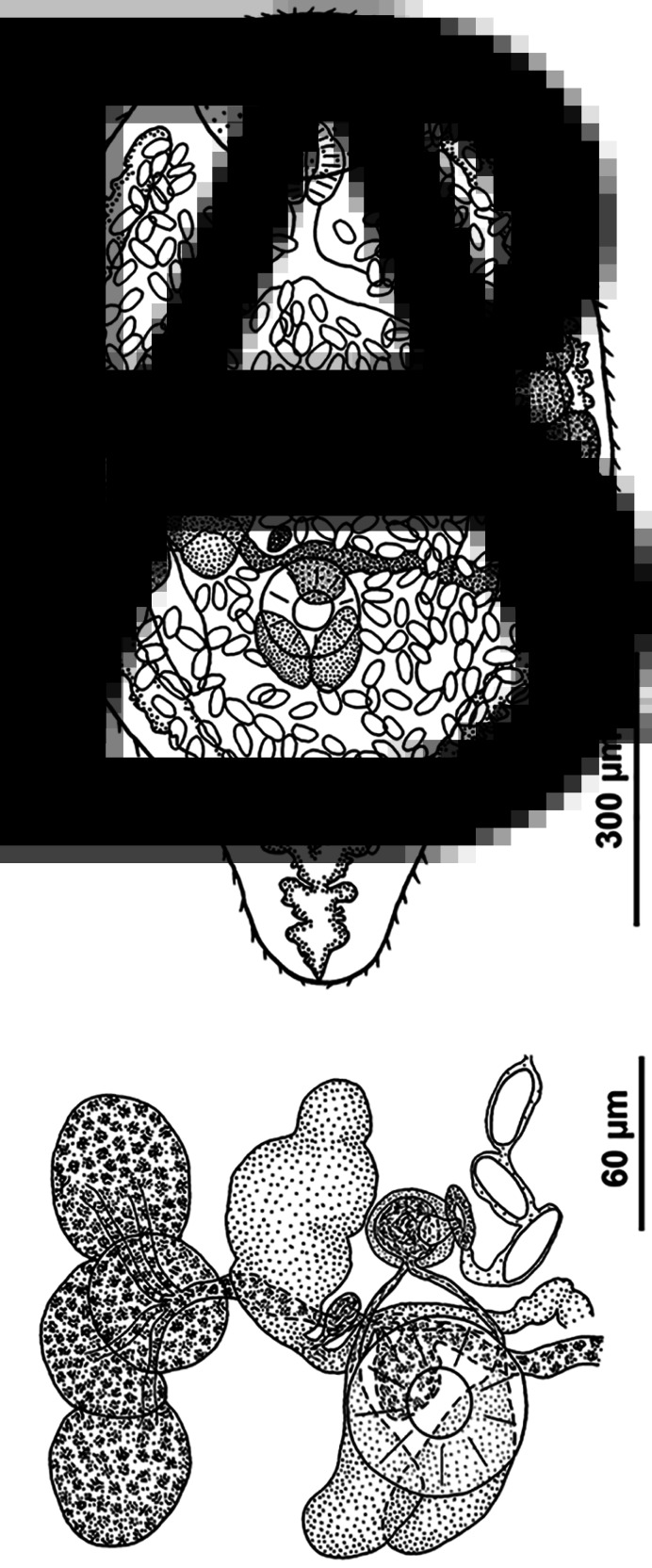
Sexual adult of *R. keimahuri*: general view from ventral side (A) and arrangement of ovary, vitellaria, genital complex and testes in relation to ventral sucker (ventral view) (B).

*Renicola keimahuri* Yamaguti, 1939 (Table 2, Figs 2C and 5)

Representative slides: 47 individuals on slides 3735-1–3735-10, deposited in the Collection of Helminths, section Trematoda, of the Zoological Institute of the Russian Academy of Sciences, St. Petersburg, Russia. This material represents paragenophores.

Representative DNA sequences: 28S rDNA (ON650720) and *cox1* (ON652706) (according to Table 1).

This species has been described by Yamaguti (1939) based on individuals from spectacled guillemot (*Cepphus carbo* Pallas, 1811) obtained in Japan. In our material, *R. keimahuri* was represented by adults from slaty-backed gull from the northern part of the Sea of Okhotsk. Considering the differences in the hosts and the geographic sites, we provide the description of the adult worms found in our study.

Worms small, drop-shaped. Oral sucker rounded, subterminal. Ventral sucker subequatorial, approximately 2–3 times smaller than the oral sucker. Prepharynx absent; pharynx small, overlapped anteriorly by oral sucker. Oesophagus short, caeca 2, extending into posterior third of body. Testes longitudinally oval, close together, sometimes partly overlapping, dorsal from ventral sucker. Vasa efferentia start from the anterior part of each testis, pass anteriorly and fuse to form a short vas deferens just before opening into the seminal vesicle (Fig. 5B). Seminal vesicle anterior to ventral sucker, median or slightly dextral of body midline. Ejaculatory duct short, opening into genital atrium. Seminal vesicle, a few prostatic gland cells and ejaculatory duct enveloped by fine membranous structure. Genital atrium slightly sinistral of seminal vesicle, opens ventrally with genital pore.

Ovary dextral (rarely sinistral), pretesticular, deeply lobbed. Oviduct starting from posterior part of ovary, receiving first seminal receptacle and then duct of vitelline reservoir. Ootype weakly developed, tubular, surrounded by Mehlis' gland cells. All ducts of female reproductive system mentioned above as well as seminal receptacle located dorsally of ventral sucker, at level of its anterior part or somewhat anteriorly. Laurer's canal absent. Ootype passing into uterus, which forms numerous ascending and descending loops and opens into genital atrium from behind. In mature worms uterus loops are densely packed with eggs and occupy almost all body volume except caudal end. Eggs operculate, elongate, their length approximately twice greater than width. Vitellarium lateral to caeca in middle third of body, consisting of 6–8 large follicles on ovarian side of body and 7–10 on opposite side. Transverse yolk ducts originating on each side as pair of ducts filled with yolk, fusing into single duct before joining with each other to form vitelline reservoir. Vitelline reservoir dorsal at the level of anterior part of ventral sucker or pre-acetabular. Excretory bladder Y-shaped with short stem in caudal end of body and 2 arms extending to level of pharynx. Stem and branches with distinct lateral diverticula.

## Molecular results

**Fig. 6.**
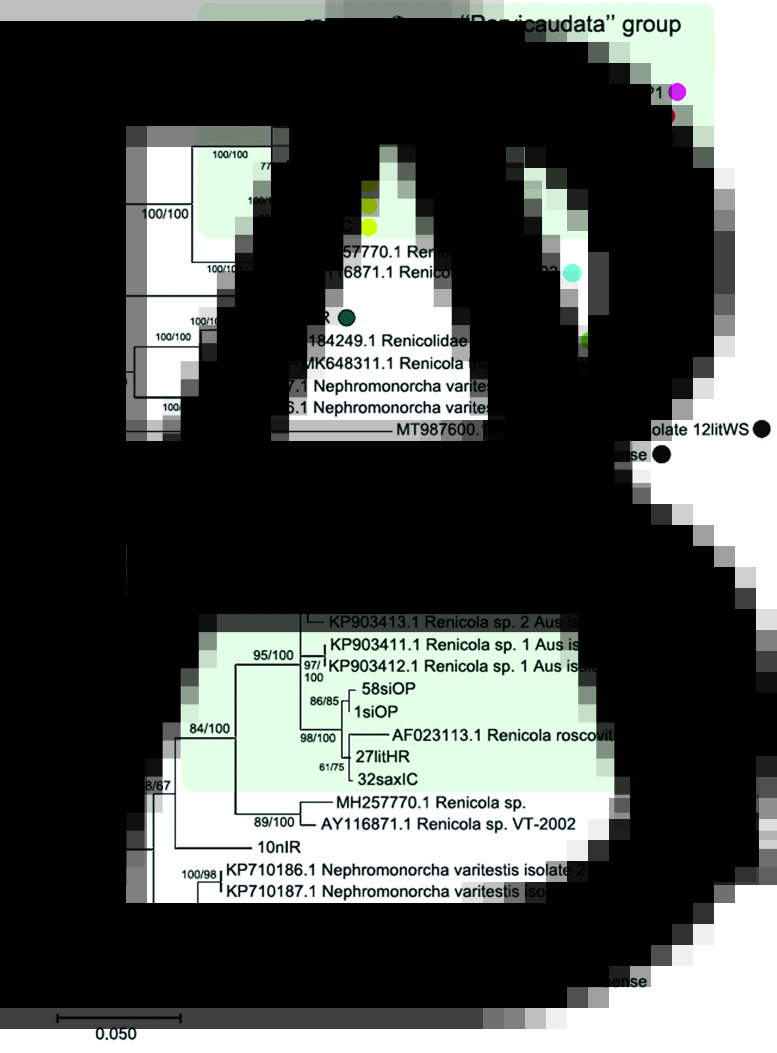
Phylogenetic relationships between *Renicola* spp. based on maximum-likelihood and Bayesian inference (BI) analyses of the D1–D3 fragment of 28S rRNA genes dataset: phylogenetic tree reconstructed with D1–D3 fragments of 28S rRNA genes (A); phylogenetic tree reconstructed with D3 fragment of 28S rRNA genes (B). Maximum-likelihood bootstrap support values inferred from 1000 replicates are followed by posterior probabilities from BI analysis. Bootstrap values followed by posterior probabilities are shown in nodes. Asterisk indicates posterior probabilities. Coloured circles indicate groups detected by ASAP tool. Yellow circles indicate *R. parvicaudatus*; yellow/black circles indicate *C. littorinae saxatilis* VIII. Light-blue ellipses indicate ‘Parvicaudata’ group.

**Fig. 7.**
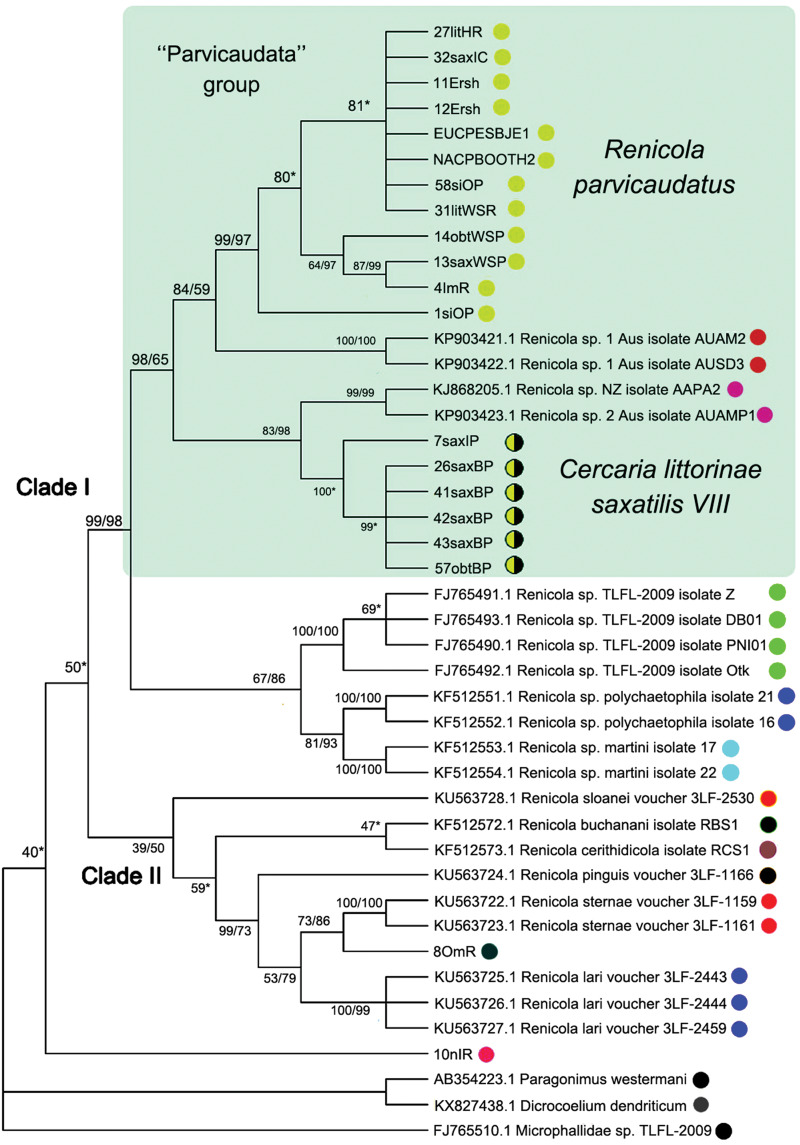
Phylogenetic relationships between *Renicola* spp. based on maximum-likelihood and Bayesian inference (BI) analyses of *cox1* gene dataset. Maximum-likelihood bootstrap support values inferred from 1000 replicates are followed by posterior probabilities from BI analysis. Asterisks indicate only bootstrap values. Coloured circles show groups detected by ASAP tool.

**Fig. 8.**
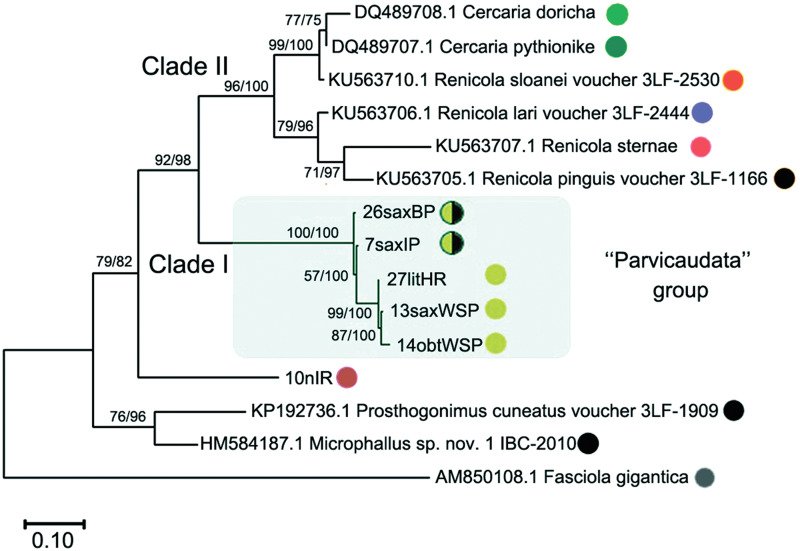
Phylogenetic relationships between *Renicola* spp. based on maximum-likelihood analysis of ITS2 dataset. Maximum-likelihood bootstrap support values inferred from 1000 replicates are followed by posterior probabilities from BI analysis. Coloured circles show groups detected by ASAP tool.

Our study generated 9 partial D1–D3 fragments of 28S rDNA (1160 bp) and 82 new mitochondrial DNA *cox1* gene sequences (313 bp) for *Renicola* spp. (Table 1). Both ML and BI analyses resulted in consensus trees with similar topologies (Figs 6–8). In addition, we obtained 6 ITS2 sequences (354–374 bp) for several isolates: 7saxIP, 10nIR, 13saxWSP, 14obtWSP, 26saxBP and 27litHR (Table 1).

In all our trees, *Renicola* spp. involved in the analysis were mostly distributed across 2 large clades (I and II). *Renicola somateriae* Belopol'skaya, 1952 (10nIR) formed a separate branch (Figs 6 and 7), which was sister to clade I in the tree based on D1–D3 fragment of 28S rRNA (Fig. 6) and sister to clade I + II in the *cox1* tree (Fig. 7). In clade I, isolates morphologically identified as *C. parvicaudata* grouped with Australian isolates of *Renicola* sp. 1 Aus O'Dwyer *et al*., 2015 and *Renicola* sp. 2 Aus O'Dwyer *et al*., 2015 into one and the same cluster, which we will refer to as the ‘Parvicaudata’ group (Figs 6 and 7). In this group, isolates tentatively identified as *C. parvicaudata* were distributed across 2 separate branches. One of the branches comprised isolates of *R. parvicaudatus sensu stricto*, and the other comprised several isolates from Iceland (7saxIP) and the Barents Sea (26saxBP, 41–43saxBP and 57obtBP), which we referred to as *C. littorinae saxatilis* VIII (see above) (Figs 6 and 7). Isolates 1siOP and 58siOP in the tree based on partial D1–D3 fragments of 28S rDNA were separate from the samples of *R. parvicaudatus* from the Netherlands (27litHR) and Iceland (32saxIC). However, genetic distances between the latter 2 samples on the one hand and 1siOP and 58siOP on the other made up 0.003 ± 0.002 and 0.004 ± 0.002, respectively, and were indistinguishable from the distance within the pooled group of these isolates, 0.003 ± 0.001 (Table S1b and S1d). The average interspecific genetic divergence amongst *Renicola* spp. ranged from 0.015 ± 0.005 (*C. littorinae saxatilis* VIII/*Renicola* sp. 2 Aus) to 0.166 ± 0.011 (*R. keimahuri*/*Renicola* sp. 2 Aus) (Table S1a).

*Renicola* sp. 2 Aus was a sister species to *C. littorinae saxatilis* VIII, while *Renicola* sp. 1 Aus was closer to *R. parvicaudatus* (Fig. 6). The genetic distance between the group of isolates of *R. parvicaudatus* and *C. littorinae saxatilis* VIII, calculated based on partial D1–D3 fragments of 28S rDNA, made up 0.028 ± 0.005, which corresponds to the interspecific level for *Renicola* (Table S1a). An analysis in ASAP also showed that the differences between *R. parvicaudatus* and *C. littorinae saxatilis* VIII corresponded to the interspecies level (Fig. 6, coloured circles). Thus, *C. littorinae saxatilis* VIII should be considered as a cryptic species relative to *R. parvicaudatus*. Our analysis also confirmed that *Renicola* sp. 1 Aus and *Renicola* sp. 2 Aus were independent species. Isolates of intramolluscan stage *Renicola* sp. Huston *et al*., 2018 (MH257770.1) found in the cerithiid gastropod *Clypeomorus batillariaeformis* Habe and Kosuge, 1966 (see Huston *et al*., 2018) and *Renicola* sp. VT-2002 (AY116871.1) from Eurasian curlew *Numenius arquata* (Linnaeus, 1758) (see Olson *et al*., 2003) (Fig. 6) formed a sister branch to the ‘Parvicaudata’ group in the tree based on partial D1–D3 fragments of 28S rDNA.

*Renicola keimahuri* (8OmR) was placed in clade II. Within this clade, it was a sister taxon to Renicolidae sp. VVT-2000 (AF184249), the sequence of renicolid intramolluscan stages from the marine gastropod *Cerithium vulgatum* Bruguière, 1792 sampled near Corsica (Tkach *et al*., 2001), in the tree based on partial D1–D3 fragments of 28S rDNA (Fig. 6). These 2 species together with *Renicola thapari* Caballero, 1953 formed, within clade II, a sister group to *Nephromonorcha varitestes* Patitucci *et al*., 2015, the only member of the genus *Nephromonorcha* represented in GenBank.

We involved in the analysis of a short fragment of 28S rRNA gene obtained from the isolate identified by Litvaitis and Rohde (1999) as *R. roscovitus* (AF023113), as it was the only marker available in GenBank for this species (Fig. 6B). The support of the branches decreased, and ASAP analysis became impossible because the branches were too short and the programme sorted all the samples into 2 groups only. However, the main clades remained unchanged in the resulting tree. The genetic distance between *R. roscovitus* (AF023113) and isolates of *R. parvicaudatus* by the shortened fragment of 28S rRNA gene made up 0.018 ± 0.007, which is equivalent to the intraspecific level (0.011 ± 0.004) (Table S1c and S1e).

In contrast to 28S rRNA gene, there are numerous nucleotide sequences of renicolids for *cox1* in GenBank. In our *cox1* phylogenetic tree, the species of the ‘Parvicaudata’ group formed a separate branch within clade I. A sister branch was represented by renicolid xiphidiocercaria species from New Zealand [*Renicola* sp. Martorelli *et al*., 2008 (FJ765490–FJ765493)] and North America (*Renicola* sp. ‘martini’ Hechinger and Miura, 2014 and *Renicola* sp. ‘polychaetophila’ Hechinger and Miura, 2014) (Fig. 7).

The phylogenetic reconstruction and the analysis in ASAP showed that groups of isolates of *R. parvicaudatus* and *C. littorinae saxatilis* VIII diverged (Fig. 7). Intragroup *p*-distances in these 2 groups varied from 0.003 ± 0.003 to 0.016 ± 0.007, while the intergroup distance made up 0.106 ± 0.016. This corresponds to the interspecific genetic divergence, which, as estimated by *cox1*, ranged amongst *Renicola* spp. from 0.094 ± 0.016 (*R. parvicaudatus*/*Renicola* sp. 1 Aus) to 0.291 ± 0.025 (*R. somateriae/Renicola sternae* Heneberg *et al*., 2016) (Table S2a). The group of *R. parvicaudatus* contained all samples from NEA, NWA and NP, including those tentatively identified (based on the colour of sporocysts) as *R. roscovitus* (11 Ersh and 12 Ersh).

Similarly to the tree based on D1–D3 fragment of 28S rRNA, the Australian species *Renicola* sp. 1 Aus in the *cox1* tree appeared as a sister to *R. parvicaudatus*, while *C. littorinae saxatilis* VIII together with *Renicola* sp. 2 Aus and *Renicola* sp. NZ O'Dwyer *et al*., 2014 formed a sister clade to them. *P*-distances between *Renicola* sp. 2 Aus and *Renicola* sp. NZ (0.035 ± 0.01) corresponded to intraspecific genetic diversity (Table S3, pair distances, Table S2b), and an analysis in ASAP did not show them to be separate species, either. Within clade II, *R. keimahuri* (8OmR) was closest to *R. sternae* and *Renicola lari* Timon-David, 1933, but *p*-distance between the former species and the latter 2 species (0.121 ± 0.018 and 0.125 ± 0.018, respectively, Table S2a) corresponded to the interspecific level. These 3 species were also distinct based on ASAP (Fig. N2, coloured circles).

In the tree based on ITS2 fragment of 28S rRNA (Fig. 8), *Cercaria doricha* Rothschild, 1935 and *Cercaria pythionike* Rothschild, 1935 belonged to the Renicolidae, grouping with representatives of clade II according to D1–D3 28S rDNA and *cox1* phylogenetic trees. The analysis in ASAP showed that *C. doricha* and *C. pythionike* were separate species, closest to *Renicola sloanei* but distinct from it. Genetic distances between *C. doricha* and *C. pythionike* also corresponded to the interspecific level (0.026 ± 0.009, Table S4). *Renicola parvicaudatus* and *C. littorina saxatilis* VIII diverged in the ITS2 tree, while the genetic distance between them based on this rDNA fragment made up 0.044 ± 0.010, which corresponds to the interspecific level (Table S4).

**Fig. 9.**
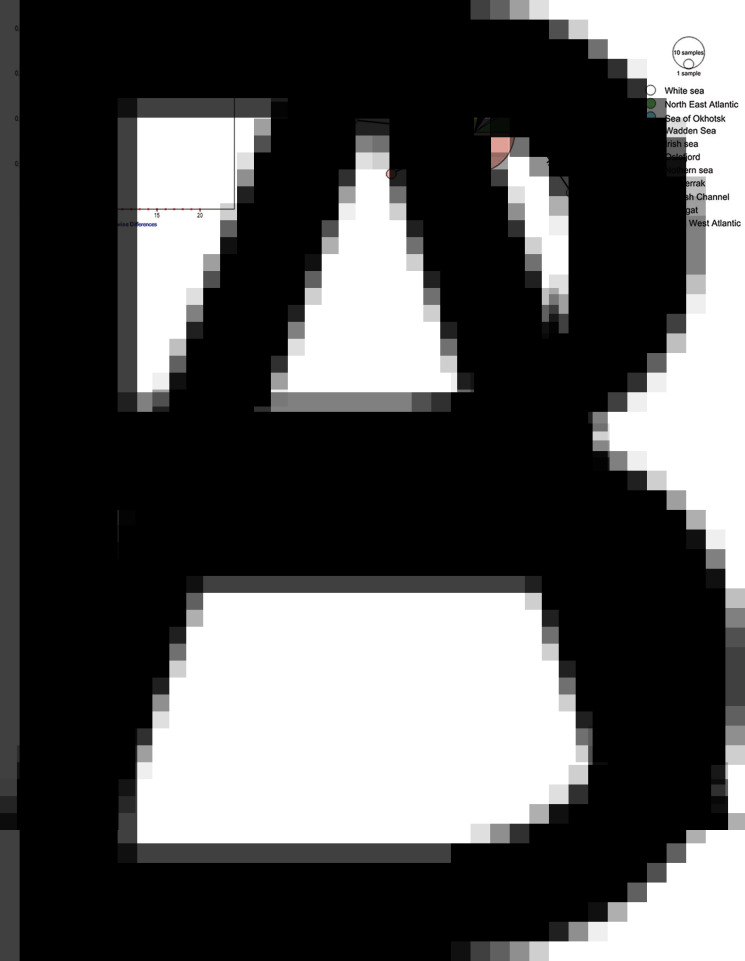
Mismatch distribution based on *cox1* haplotypes (A) and a median joining haplotype network for *R. parvicaudatus* (B) based on *cox1* gene sequences. Solid lines in mismatch distribution graph show observed frequencies, dashed lines show expected frequencies. Colours in haplotype network indicate sampling regions, circle size is proportional to sample size. Hatch marks represent nucleotide substitutions. Black dots represent missing haplotypes.

To study the history and the structure of *R. parvicaudatus* population, we calculated the mismatch distribution and constructed a haplotype network (Fig. 9). Mismatch distribution showed low pairwise differences and was skewed unimodal (Fig. 9A). We detected 10 haplotypes, which were arranged in a ‘star’ network (Fig. 9B). Most isolates represented the main haplotype, except the isolates from the White Sea and one of the Sea of Okhotsk isolates (Fig. 9B). The latter was separated from the dominant haplotype by 4 substitutions. An additional haplotype is conjectured to be present between the White Sea haplotypes and the dominant one (black dot, Fig. 9B). The Tajima's *D* neutrality test resulted in –2.239 (*P* < 0.01).

## Remarks

### Adults of *R. parvicaudatus*

Adult *Renicola* from gulls in Iceland matched in all the molecular markers used in our study the intramolluscan stages of *C. parvicaudata* both from periwinkles collected at the Atlantic coast of Europe and North America and from periwinkles collected in the Sea of Okhotsk (Figs 6–8). In particular, they matched the cercariae *C. parvicaudata* collected from snails *L. littorea* in the Woods Hole region (11G′ Ersh and 12D′ Ersh), that is, from the same place and the same molluscan host as in the first description by Stunkard and Shaw (1931). Morphologically these adults matched the adults of *R. roscovitus* described by Werding (1969) (Table 2). Since the description of *C. parvicaudata* Stunkard and Shaw, 1931 was published before that of *C. roscovita* Stunkard, 1932, in accordance with the International Code of Zoological Nomenclature (ICZN, 1999) (23.1. Statement of the Principle of Priority) a valid species name would be *R. parvicaudatus* (Stunkard and Shaw, 1931) nov. comb. The use of the name *R. roscovitus* should be discontinued until the clarification of the status of *C. roscovita* (see Remarks on cercariae). Three adult worms found in a slaty-backed gull *L. schistisagus* obtained in the north of the Sea of Okhotsk also agreed with the description of adult *R. parvicaudatus* (Табл. 2, Fig. 2A and B). In addition, snails *L. sitkana* from that area (Nagaeva Bay) harboured sporocysts and cercariae (isolates 1siOP and 58sitO) that corresponded to *C. parvicaudata* from the European periwinkles based on molecular and morphological characters.

The adults (but not the cercariae!) described by Stunkard (1964) as *R. thaidus* should also be synonymized with *R. parvicaudatus.* To note, the synonymy of *R. thaidus* and *R. roscovitus* (=*R. parvicaudatus*) has been suggested by Werding (1969). The adults of *R. parvicaudatus* and those of *R. thaidus* are similar morphometrically (Table 2). Other similarities are the location of vitellaria in the body of the worms (in 2 symmetrical lateral groups in the posterior body part, from the base of the tail to approximately the level of anterior border of the ovary) as well as a lobate ovary and 2 oval testes.

The cercaria described by Stunkard (1964) from Woods Hole region as the larva of *R. thaidus* is common at the European coast of the NA in molluscs *Nucella lapillus* (Linnaeus, 1758) (see Galaktionov and Skírnisson, 2000). This larva is markedly different from the species of the ‘Parvicaudata’ group both morphologically (Galaktionov and Skírnisson, 2000) and in molecular markers used in this study (Figs 6–8). Skírnisson *et al*. (2002–2003–2003) showed with the use of the ITS1 rDNA marker sequence that this cercaria is the larva of adult worms *R. somateriae* parasitizing in the kidneys of common eider (*Somateria mollissima* Linnaeus, 1758) (this conclusion was supported by the analysis of 28S rDNA and *cox1* mitochondrial DNA sequences, our unpublished data). The adults of *R. somateriae* are strikingly different from those of *R. parvicaudata* as their vitellaria stretch laterally in 2 symmetrical rows from approximately the level of the pharynx to the tail.

Stunkard (1964) raised adults of *R. thaidus* in a young herring gull by feeding it on mussels experimentally infected with metacercariae of *R. thaidus*, but failed to raise them in ducklings of common eider. Apparently, the experiments went wrong at some stage (most probably, some of the mussels had natural infection with metacercariae of *R. roscovita*). Later Stunkard (1971) repeatedly tried to infect gulls *L. argentatus*, cormorants, chicks, ducklings and laboratory mammals with metacercariae of *C. parvicaudata* from periwinkles and mussels collected near Woods Hole, but unsuccessfully. Therefore, he assumed that *R. roscovitus* and *C. parvicaudata* were different species and that while the former, in accordance with Werding (1969), used gulls as the definitive host, the latter could use some sandpipers (Stunkard, 1971). It is difficult to say why Stunkard's experiments with infection of the birds were unsuccessful, but our results unequivocally indicate that the definitive hosts of *R. parvicaudatus* are gulls.

In principle, there may be another species that should be made synonymous with *R. parvicaudatus*: *Renicola murmanicus* Belopol'skaya, 1952 described by Belopol'skaya (1952) from gulls in Eastern Murman (Barents Sea). The adults of these 2 species are morphologically and morphometrically identical (Table 2). We do not synonymize them yet because the adults of *R. murmanicus* have been registered in the same region (Barents Sea, Eastern Murman) where, according to our observations, the periwinkles are infected only with intramolluscan stages of the cryptic species *C. littorinae saxatilis* VIII. It is possible that they are the life-cycle stages of one and the same species. In that case, it should be referred to as *R. murmanicus* and considered a cryptic species relative to *R. parvicaudatus*.

### Cercariae of *R. parvicaudatus* and *C. littorinae saxatilis* VIII

Morphologically, the cercaria *R. parvicaudatus* described in our study completely matches the cercariae of *C. parvicaudata*, whose intramolluscan stages have been described by Stunkard and Shaw (1931) from snails *L. littorea* in the Woods Hole region (north of the USA East Coast). Later, Stunkard (1950) supplemented the description and added *L. saxatilis* and *L. obtusata*, also from the Woods Hole region, to the list of the first intermediate hosts. *Cercaria roscovita* has been described by Stunkard (1932) from *L. saxatilis* from Roscoff (France, Atlantic coast). Intramolluscan stages of *C. parvicaudata* and *C. roscovita* are barely distinguishable from each other. Stunkard (1950), when differentiating between these 2 species, noted that ‘except for the difference in colour of the daughter sporocysts, the 2 species are almost identical’. However, the colour of the parthenitae cannot be considered as a reliable character for species differentiation (Werding, 1969; Galaktionov and Skírnisson, 2000). It depends on the infection age: young groups of sporocysts of *R. parvicaudatus* (infection of the current year) in periwinkles at the White Sea are white, while old groups that have overwintered in the molluscan host are lemon yellow (Nikolaev *et al*., 2021). Nadakal (1960) has shown that daughter sporocyst and redial colour is determined by the presence of *β*-carotene accumulated both in the molluscan tissues and in the parasites. The source of carotenoids in the molluscan organism is the alga the molluscs feed on. In case of the renicolids in our material, it was not so much the sporocysts that were coloured but the layers of molluscan tissues between them. Our analysis of *cox1* sequences of the sporocysts from lemon-coloured pseudo-tumours [*C. roscovita* in accordance with Stunkard (1950)] and from orange-coloured ones [*C. parvicaudata* in accordance with Stunkard (1950)] showed that they belonged to the same species, which we refer to as *R. parvicaudatus*. To conclude, differences in the colour of the sporocysts (or, rather, in the colour of the surrounding host tissues) cannot be considered as a diagnostic character.

There is 1 character that remains to be discussed, and it is the number of penetration gland cells. Stunkard (1950) indicated that the cercariae of *C. parvicaudata* had 6 pairs of penetration gland cells, while cercaria of *C. roscovita* had ‘several’ (Stunkard, 1932, 1950). This character was later used for differentiating the cercariae of these 2 species by James (1968*a*, 1969). It is difficult to count the penetration gland cells in renicolid cercariae, because the distal parts of their ducts are extremely narrow while the nuclei-containing cell bodies are obscured by numerous cystogenous gland cells. Werding (1969) noted that the number and the exact location of penetration gland cells in the cercariae described by him as *R. roscovitus* could not be determined. This may be the reason why Stunkard (1950) did not include this character into the list of characters differentiating the 2 species of cercariae under consideration.

However, the number of penetration gland cells is mentioned in the identification keys by James (1968*a*), who differentiated the cercariae of *C. parvicaudata* and those of *C. roscovita* based on sporocyst colour and the number of penetration gland cells. It is noteworthy that the cercaria of *C. roscovita* is said to have ‘numerous’ gland cells. In the drawing of a cercaria of this species from *L. saxatilis* in Cardigan Bay, Wales (UK), 15–17 pairs of penetration gland cells can be counted, whose external pores form 2 longitudinal rows on either side of the stylet (Fig. 77, p. 301, James, 1969). This drawing disagrees with our data and with the drawing of a cercaria of *R. roscovitus* in Werding (1969), in which the ducts of the penetration gland cells open in 2 compact groups near the anterior edge of the oral sucker in the area of the stylet, that is, exactly as they do in *C. parvicaudata*.

In our opinion, it was *C. parvicaudata* that Werding (1969) studied, not *C. roscovita* described by James (1969). This opinion is supported by the fact that Werding (1969) worked with intramolluscan stages from *L. littorea*, while *C. roscovita* has been reported only from *L. saxatilis* and *Melarhaphe neritoide*s (Linnaeus, 1758) (Syn. *Littorina neritides*) (Stunkard, 1932, 1971; James, 1968*b*, 1969). The region where Werding (1969) collected his material is the same as the region where Litvaitis and Rohde (1999) worked: the coast of Germany, including Isle of Sylt (Wadden Sea). Moreover, the sequence of short 28S DNA fragment of *R. roscovitus* (AF023113) from Litvaitis and Rohde (1999) matched the sequences that we obtained for *C. parvicaudata* (Fig. 6B). Snails *L. littorea* from the North Sea coast (Texel Island, the Netherlands) surveyed in our study were infected only with intramolluscan stages of *C. parvicaudata*, as supported by molecular data (Figs 6A and 7).

In addition to *C. parvicaudata* and *C. roscovita*, cercariae of 3 other renicolid species are recorded in periwinkles in NA: *Cercaria emasculans* Pelseneer, 1906, *Cercaria brevicauda* Pelseneer, 1906 and *C. littorinae saxatilis* VI Sannia and James, 1977 (James, 1968*a*; Sannia and James, 1977). They differ from *C. parvicaudata* and *C. roscovita* in morphometric characteristics, the shape of the stylet, the number of penetration gland cells and the position of their ducts in the larval body. *Cercaria littorinae saxatilis* VI, which has been described from *L. saxatilis* in the north of Iceland (Eyjafjördur) (Sannia and James, 1977), is strikingly different from the larvae of the other species, because it has only 1 pair of penetration gland cells. We did not find any cercariae of this species in the south-western Iceland though we dissected more than 10 000 individuals of *L. saxatilis* and *L. obtusata* in the course of our surveys; we registered only intramolluscan stages of *C. parvicaudata* (Galaktionov and Skírnisson, 2000; Skírnisson and Galaktionov, 2002; K. V. Galaktionov, personal observations) and, as molecular analysis showed, those of a cryptic species *C. littorinae saxatilis* VIII.

Cercariae of *Renicola* sp. NZ, *Renicola* sp. 1 Aus and *Renicola* sp. 2 Aus from Australian and New Zealand *Austrolittorina* spp., which make up the ‘Parvicaudata’ group together with *R. parvicaudatus* and *C. littorinae saxatilis* VIII, differ from the latter 2 species genetically as well as in the number of penetration gland cells (5 pairs), number and position of large spines in the suckers and of sensory papillae on the body surface (chaetotaxy) (O'Dwyer *et al*., 2014, 2015; Denisova and Shchenkov, 2020).

Summing up, our molecular and morphological studies indicate that *R. parvicaudatus* is the most common species among the renicolid intramolluscan stages in snails *Littorina* spp. at the Atlantic coast of Europe and North America. There are no credible findings of *C. roscovita* in this area. Werding (1969) suggested to synonymize these 2 species under the name of *R. roscovitus* (as noted before, he dealt with *R. parvicaudatus*). Nevertheless, it is premature to synonymize *C. roscovita* Stunkard, 1932 with *R. parvicaudatus* because: (1) a cercaria with numerous penetration gland cells, minutely described by James (1969), should be attributed to *C. roscovita* Stunkard, 1932 and (2) Denisova and Shchenkov (2020) found that the number and position of the sensory receptors on the body of cercariae *C. parvicaudata* from *L. littorea* at the White Sea were different from those of *C. roscovita* from *L. saxatilis* near Roscoff (Richard, 1971), that is, the same snail species and the same site from which this larva was first described by Stunkard (1932). It cannot be ruled out that the species *R. roscovitus* does exist, and its transmission is implemented further southwards in the Atlantic (e.g. British Isles, France). At the same time, intramolluscan stages of *R. roscovitus* parasitize only snails *L. saxatilis* and *M. neritoide*s, while *R. parvicaudatus* is found in *L. littorea* and, more rarely, in *L. saxatilis* and *L. obtusata*. To note, the analysis of *cox1* sequences of the isolates from the Atlantic coast of France including the vicinity of Roscoff (EUCPTROUV1, EUCPTROUV2, EUCPTROUV3, EUCPMINDI1, EUCPMINDI2, EUCPMINDI3, EUCPMINDI4 and EUCPMINDI5 – Table 1) did not reveal the presence of any species different from *R. parvicaudatus*. Whether or not *R. roscovitus* is a true species can only be established in integrative morphological and molecular studies of intramolluscan stages of renicolids in periwinkles from the British Isles and the Atlantic coast of France.

### Renicola keimahuri

Adult worms of the second species isolated from gulls *L. schistisagus* from the Sea of Okhotsk in our study (isolate 8OmR) morphologically correspond to *R. keimahuri* described by Yamaguti (1939) from spectacled guillemot *C. carbo* in Japan. They are somewhat smaller than the worms described by Yamaguti (1939) (Table 2), which may be associated with the host-induced variability. Leonov *et al*. (1963) recorded *R. keimahuri* in larids in Kamchatka: slaty-backed gull (*L. schistisagus*), black-legged kittiwake [*Rissa tridactyla* (Linnaeus, 1758)], common tern [*Sterna hirundo* (Linnaeus, 1758)] and Aleutian tern (*Onychoprion aleuticus* Baird, 1869). This broad range of hosts may indicate that we deal with a complex of close or cryptic species. Detailed morphological and molecular studies are needed to prove or disprove this hypothesis. To note, this hypothesis is also supported by some morphological differences of *R. keimahuri* in Leonov *et al*. (1963) from the first description by Yamaguti (1939) and the description given above: fewer vitelline follicles (4–5) and testes that do not touch each other but are spaced apart [Fig. 20, p. 151 from Leonov *et al*. (1963)]. At the same time, we examined mounted specimens of *R. keimahuri* from gulls *L. schistisagus* of Kamchatka (mounts ## 2439/Tr–2441/Tr, col. & det. Leonov) deposited in the collection of the Centre of Parasitology of the Russian Academy of Sciences and found that they fully corresponded to those described in this study.

Both by the molecular marker *cox1* and by morphological criteria *R. keimahuri* is closest to the European species *R. sternae* described by Heneberg *et al*. (2016) from common tern (*S. hirundo*) and to *R. lari* from the herring gull (*L. argentatus*) and black-headed gull (*L. ridibundus* Linnaeus, 1766) (Prevot and Bartoli, 1978). These 3 species are similar in size and morphology (Table 2). *Renicola sternae* differs from *R. keimahuri* in having separate testes lying beside the ventral sucker (but see remark in Discussion) and in somewhat greater number of follicles in the vitellaria: 6–12 on the ovarian side of body and 9–13 on the opposite side. *Renicola lari* is slightly larger than *R. keimahuri* from our material, but corresponds to the size characteristics of this species given by Yamaguti (1939). Despite their morphological similarity, *R. keimahuri*, *R. sternae* and *R. lari* are quite distinct genetically.

## Discussion

It was shown for the first time that out of all *Renicola* spp. using snails *Littorina* spp. as the first intermediate hosts in the nearshore areas of NA seas the dominant species is *C. parvicaudata*, as identified based on the combination of morphological characters. The analysis of molecular markers and morphology showed that the adults of this species are found in gulls from Iceland and the Sea of Okhotsk. This means that we successfully elucidated the life cycle of this species. According to the Code of Zoological Nomenclature (ICZN, 1999), we name it *R. parvicaudatus* (Stunkard and Shaw, 1931) nov. comb. The name *R. roscovitus* (Stunkard, 1932) Werding, 1969 and the name *R. thaidus* Stunkard, 1964 used by Stunkard (1964) for the adult worms should be considered as its synonyms.

The cercariae of *C. parvicaudata* and those of *C. roscovita* are difficult to differentiate. This circumstance gave rise to a long-lasting confusion. It started with an experimental study of Werding (1969), who identified the cercaria of the renicolid species whose life cycle he studied as *C. roscovita* and named the species *R. roscovitus*. As explained in the Remarks section above, Werding (1969) actually studied the cercariae of *R. parvicaudatus*. This means that intramolluscan stages from *Littorina* spp. identified in numerous ecological and faunistic studies as *R. roscovitus* (e.g. Lauckner, 1987; Granovitch and Johannesson, 2000; Thieltges, 2006; Thieltges and Rick, 2006; Mouritsen and Elkjær, 2020) actually belong to *R. parvicaudatus* or to its cryptic species *C. littorinae saxatils* VIII first described in this study.

### ‘Parvicaudata’ species complex: composition and phylogeography

The species of the ‘Parvicaudata’ group form a separate clade on phylograms constructed on the basis of molecular markers used in our study. All these species use intertidal snails *Littorina* spp. and *Austrolittorina* spp. (Littorinoidea, Littorinidae) as the first intermediate host. The definitive host is known only for *R. parvicaudatus*, but other species of the ‘Parvicaudata’ group probably also use gulls or other birds, such as sandpipers, that feed on nearshore invertebrates. A sister clade of the ‘Parvicaudata’ group is formed by species whose first intermediate hosts are various molluscs from the superfamily Cerithioidea, some of which belong to the family Cerithiidae (*C. batillariaeformis* Habe and Kosuge, 1966) (Cannon, 1978, 1979), some to the Batillariidae [*Zeacumantus subcarinatus* (G. B. Sowerby II, 1855)] (Leung *et al*., 2009) and some to the Potamididae [*Cerithideopsis californica* (Haldeman, 1840)] (Hechinger and Miura, 2014). This observation suggests that the formation of the ‘Parvicaudata’ group was associated with the colonization of periwinkles as the first intermediate host.

The only morphological differences between cercariae of *R. parvicaudatus* and *C. littorinae saxatilis* VIII on the one hand and the larvae of Australian-New Zealand species on the other are the number of penetration gland cells and the number and position of large spines in the suckers and the sensory papillae on the body surface (see Remarks). *Renicola* sp. NZ and *Renicola* sp. 2 Aus have some differences in the latter 2 characters and the size (O'Dwyer *et al*., 2015), but genetic divergence between them was within the species level (Tables S2b and S3, Figs 6A and 7), which means that they are likely to be morphs of the same species. At the same time, genetic differences between morphologically indistinguishable cercariae of *R. parvicaudatus* and *C. littorinae saxatilis* VIII corresponded to those between different species (Tables S1a, S2a and S4), which suggests that they are cryptic species. At the same time, *C. littorinae saxatilis* VIII is genetically closer to Australian-New Zealand species that to *R. parvicaudatus* (Tables S1a, S2a and S4, Figs 6 and 7).

To sum up, morphological differences between the renicolid cercariae may not necessarily mean that they belong to different species. By the same token, the absence of morphological differences does not prove that the cercariae are conspecific. The case of *Renicola* sp. NZ and *Renicola* sp. 2 Aus shows that subtle differences in cercarial morphology and chaetotaxy revealed with the use of SEM (O'Dwyer *et al*., 2014, 2015) are not always reliable criteria for species differentiation. These considerations strongly indicate that an integrative approach is the key to ascertaining the species status of digeneans. This approach should involve the analysis of morphological characters (preferably, of all life-cycle stages), molecular markers and the data on the larval and adult biology, host range, transmission pathways and geographic distribution (Blasco-Costa *et al*., 2016; Blasco-Costa and Poulin, 2017; Gonchar and Galaktionov, 2021, 2022).

Small genetic distances between the species of the ‘Parvicaudata’ group (Tables S1a, S2a and S4) indicate its relatively recent formation. The differentiation of *R. parvicaudatus* could be associated with the colonization of a new first intermediate host, the snail *L. (Littorina) littorea*, the only Atlantic species of the subgenus *Littorina*. Its ancestor split from its NP sister species *L. (Littorina) squalida* Broderip and G. B. Sowerby I, 1829 and colonized the NA *via* the Arctic route ca. 5.5–2.4 million years ago (Reid, 1996; Reid *et al*., 1996, 2012). This assumption is supported by the facts that *R. parvicaudatus* is the only renicolid parasitizing *L. (L.) littorea* and that it occurs in the latter more frequently than in the Atlantic periwinkles of the subgenus *Neritrema*, i.e. *L. (N.) saxatilis* and *L. (N.) obtusata* (our data). Intramolluscan stages of *R. parvicaudatus* have never been registered in *L. (L.) squalida* in NP (Tsimbaljuk *et al*., 1978; Rybakov, 1983; our data), and out of all the Pacific *Neritrema*, only *L. (N.) sitkana* serves as their host, and only rarely (Tsimbaljuk *et al*., 1978; our data). Intramolluscan stages of the other species of the ‘Parvicaudata’ group develop in Atlantic periwinkles of the subgenus *Neritrema* (*C. littorinae saxatilis* VIII as well as *C. emasculans*, *C. brevicauda* and *C. littorinae saxatilis* VI, which most likely also belong to this group) or in Australian *Austrolittorina* spp. (James, 1968*a*, 1969; Sannia and James, 1977; O'Dwyer *et al*., 2014, 2015; our data).

The star-like patterns in cox1 haplotype network for *R. parvicaudatus* suggest a low geographic structure (Fig. 9B). Thus, we may conclude that *R. parvicaudatus* is represented by a single population throughout its Holarctic range (so far this species has not been detected at the Pacific coast of North America). The widespread haplotype positioned at the centre of the network can be considered as the ancestral one (Jenkins *et al*., 2018). The other haplotypes, which are linked to this dominant haplotype by a single mutational step or a few steps, are the result of recent mutation events. Unimodal mismatch distribution (Fig. 9A) and significant negative value of the Tajima's *D* indicate a bottleneck event, possibly dating from the last glacial maximum (LGM). During LGM, the transmission of *R. parvicaudatus* may have persisted in one of the NEA glacial refugia, where periwinkles, including *L. littorea*, and seabirds, including gulls, were concentrated. The refugium in question could be one of the southern refugia near the Iberian Peninsula and the British Isles, where *L. littorea* survived during LGM (Maggs *et al*., 2008; Blakeslee *et al*., 2021). Thus, contraction into a single refugium appears to have resulted in a strong bottleneck for both the ancestral *L. littorea* (Blakeslee *et al*., 2021) and its parasite, *R. parvicaudatus*.

If that is the case, the post-LGM expansion of *R. parvicaudatus* proceeded along the Atlantic coast of Europe, following the advance of their first intermediate hosts. The incursion into the NWA may have occurred after *L. littorea* was introduced into this region from the NEA. This introduction was human-mediated and tentatively dates back to the 19th century (Blakeslee *et al*., 2008, 2021). The fact that almost all NWA haplotypes of *R. parvicaudatus* coincide with the dominant haplotype (Fig. 9B) can be interpreted as a further evidence of a fairly recent introduction of *L. littorea* into the NWA. Alternatively, natural trans-Atlantic migration with another first-intermediate periwinkle, *L. obtusata*, may have transported *R. parvicaudatus* to the NWA. This is because this periwinkle appears to have recolonized the NWA following glacial retreat *via* a stepping stone migration across NA islands (Wares and Cunningham, 2001).

Two circumstances explain the fact that *R. parvicaudatus* has a broad geographical distribution and, at the same time, its *cox1* haplotypes are identical or very similar in different parts of its range. Firstly, the definitive hosts of this parasite are highly mobile migrating birds such as gulls, and secondly, the life span of adult worms in them is very long. Gulls that breed at high latitudes (e.g. *L. argentatus*, *Larus fuscus* Linnaeus, 1758, *Larus canus* Linnaeus, 1758, *Larus glaucescens* Naumann, 1840, *Larus glaucoides* Meyer, 1822, *L. schistisagus*) make long seasonal migrations along the coasts of Europe and North America (Helberg *et al*., 2009; Newton, 2010; Hallgrimsson *et al*., 2012; Klaassen *et al*., 2012; Davis *et al*., 2016; Anderson *et al*., 2020). White-headed gulls associated with coastal habitats, such as *L. argentatus* and *L. canus*, which have a circumpolar distribution, were shown to have a limited population genetic subdivision among northern Arctic populations (Sonsthagen *et al*., 2012). This observation indicates that there is an intense genetic exchange between the populations of these birds owing to their migratory activity. Some individuals, apparently, are even capable of making trans-Arctic flights. Otherwise, the coincidence of *R. parvicaudatus* haplotypes in NA and NP would be difficult to explain. Trans-Arctic flights are known for Arctic-breeding seabirds (Clairbaux *et al*., 2019) and have recently been reported for a larid bird, the black-legged kittiwake (*R. tridactyla*) (Ezhov *et al*., 2021). Another option is the transfer of the parasite by birds from the Atlantic coast of North America to the Pacific coast, and from there to the coast of North Asia.

The snag of both hypothetical variants of the trans-Arctic transfer of *R. parvicaudatus* is the absence of its first intermediate hosts, the periwinkles, at the coasts of the Siberian seas and at the Arctic coast of North America (Arctic coast of Alaska and the Canadian Arctic Archipelago) (Reid, 1996). Only long-living helminths such as renicolids can endure such a long flight. According to Werding (1969), the lifespan of *R. parvicaudatus* (*R. roscovitus* in Werding's article) in the final host is at least 7 months. The fact that one of the haplotypes from the Sea of Okhotsk coincides with the dominant one indicates that there is an ongoing exchange between NA and NP parts of the *R. parvicaudatus* population (Fig. 9B**)**. It may be associated with the warming of the Arctic, which opens opportunities for trans-Arctic bird migrations (Clairbaux *et al*., 2019; Ezhov *et al*., 2021). Another haplotype of *R. parvicaudatus* from the Sea of Okhotsk is significantly different from the Atlantic one (Fig. 9B**)**, possibly indicating some degree of isolation between the NP and the NA part of the parasite's population. Another evidence of the possibility of some local differentiation within the population of *R. parvicaudatus* is the fact that all the haplotypes from the White Sea are different from the dominant one (Fig. 9B**)**.

In our opinion, it is premature to hypothesize about the ways of geographical expansion of other species of the ‘Parvicaudata’ group since genetic data are limited and we do not know the actual number of species constituting the group, the array of their second intermediate and definitive hosts and their ranges. The establishment of the Australian-New Zealand species was probably associated with the colonization of the local *Austrolittorina* spp., the incursion of the ancestral species being ensured by migrating birds. Considering that in our phylograms the Australian species *Renicola* sp. 1 Aus is sister to the Holarctic *R. parvicaudatus*, while the group comprising Australian-New Zealand *Renicola* sp. 2 Aus and Renicola sp. NZ is sister to the NA *C. littorinae saxatilis* VIII, we can assume that Australia and New Zealand were colonized as a result of 2 putative independent events.

### Notes on taxonomy and phylogeny of renicolids

Two large clusters can be seen in the phylograms constructed on the basis of the molecular markers used in our study (Figs 6 and 7). *Renicola parvicaudatus* falls into cluster I, while *R. keimahuri* falls into cluster II. There are considerable morphological differences between species in cluster I and those in cluster II. Moreover, these differences are pronounced at all life-cycle stages. The adults differ in the position of vitellaria and testes, which is considered as an important taxonomical character in renicolids (Wright, 1956, 1957; Odening, 1962; Sudarikov and Stenko, 1984; Gibson, 2008). The adults of *R. parvicaudatus* (the only species from cluster I for which adults have been described) have vitellaria in the posterior body part and separate, non-contiguous testes. At the same time, in all adults from cluster II described so far vitellaria are located lateral to the caeca in the middle third of the body, which is a characteristic feature of species from the *pinguis*-group in accordance with Wright (1957) and Odening (1962). Testes are contiguous [*R. thapari*, *R. sloanei* Wright, 1954, *Renicola pinguis* (Mehlis in Creplin, 1846) Cohn, 1904, *R. lari* and *R. keimahuri* (Yamaguti, 1939; Caballero, 1953; Wright, 1954; Prevot and Bartoli, 1978; Rubio-Godoy *et al*., 2011; Matos *et al*., 2019; this study)] or fused to form a single mass, which is a diagnostic character of *Nephromonorcha* (Gibson, 2008). *Renicola sternae* is an exception: Fig. 2b (p. 1601) in Heneberg *et al*. (2016) shows testes lying laterally of the ventral sucker. To note, however, Fig. 2a (p. 1601) in Heneberg *et al*. (2016) shows a similar position of testes in *R. lari*, which is at odds with the description of Prevot and Bartoli (1978). This means that the position of testes in *R. sternae* and *R. lari* should be verified.

*Nephromonorcha varitestis*, the only species of the genus for which molecular data are available (Patitucci *et al*., 2015), forms an independent branch within cluster II in our phylograms. This finding supports the validity of the genus *Nephromonorcha*. The tendency towards contingence and subsequent merging of the testes seems to be characteristic of species in cluster II. Partial merging of contiguous testes is noted for *R. lari* (Prevot and Bartoli, 1978), and we observed similar pictures in *R. keimahuri*. At the same time, an incomplete merging of the testes into a common mass has been described in some individuals of *Nephromonorcha ralli* Byrd and Heard, 1970 and *N. varitestis* (Byrd and Heard, 1970; Patitucci *et al*., 2015). In addition to the species involved in our analysis, testes contiguous near the ventral sucker have been noted in some other renicolids, e.g. *Renicola wright* Odening, 1962, *Renicola pelecani* Wright, 1954, *Renicola fischeri* Odening, 1962, *Renicola pseudosloanei* Odening, 1962, *Renicola hayesannieae* Byrd and Kellogg, 1972, *Renicola pollaris* Kontrimavitschus and Bachmet'eva, 1960, *Renicola glacialis* Riley and Owen, 1972 and *Renicola williamsi* Munyer and Holloway, 1990 (Wright, 1954, 1956; Kontrimavitschus and Bachmet'eva, 1960; Odening, 1962; Byrd and Kellogg, 1972; Riley and Owen, 1972; Munyer and Holloway, 1990). The emergence of this character in the course of morphological evolution of adult renicolids could be determined by the pressure on the testes from the eggs in the uterus loops expanding in posterior–lateral direction and could occur independently in different phylogenetic lineages of this taxon. For instance, contiguous or even merging testes are noted in *Renicola philippensis* Stunkard *et al.*, 1958 (Stunkard *et al*., 1958) and *R. hayesannieae* (Byrd and Kellogg, 1972). At the same time, vitellaria in these species are located in the posterior-lateral part of the body, as in *R. parvicaudatus* from cluster I. Apparently, the determination of the taxonomic ‘weight’ of all these characters requires a more complete molecular phylogeny of renicolids involving a greater number of species differing in the structure and position of testes and vitellaria.

Structural differences between the species from the 2 clusters identified in our molecular phylogenies concern not only adults but also cercariae. Cercariae of all species from cluster I look like typical xiphidiocercariae: small size (body and tail each approximately 150–250 *μ*m long), stylet, 1–6 pairs of penetration gland cells (rarely more), excretory formula 2[(3 + 3 + 3) + (3 + 3 + 3)] = 36, main collecting tubes join the stem of the excretory bladder, simple tail (Hechinger and Miura, 2014; O'Dwyer *et al*., 2014, 2015). Xiphidiocercariae are also known for some renicolids for which molecular data are lacking, e.g. *Cercaria opaca* Holliman, 1961, *Cercaria caribbea* XXXII Cable, 1956, *C. caribbea* XXXIII Cable, 1956 (Cable, 1956; Holliman, 1961). The cercaria of *R. somateriae* (isolate 10nIR, syn. *R. thaidus* Stunkard, 1964), which is sister to I or I + II in the phylogenetic trees (Figs 6 and 7), also looks like a typical xiphidiocercaria (Stunkard, 1964).

Cercariae of *Renicola buchanani* (Martin and Gregory, 1951) and *Renicola cerithidicola* Martin, 1971 in clade II have the same general appearance but lack the stylet (Martin and Gregory, 1951; Martin, 1971). In *R. lari*, which is similar to *R. keimahuri*, cercaria, besides lacking the stylet, also have a well-developed excretory bladder with a short stem and arms extending to the anterior end of the body and carrying numerous lateral diverticula (Prevot and Bartoli, 1978). In addition, though the excretory formula remains the same, the main collecting tubes join not the stem but the arms of the excretory bladder. Prevot and Bartoli (1978) considered cercariae *R. lari* and a similar *C. caribbea* VIII Cable, 1956 as a transitional morphotype to the typical cercariae of Rhodometopa group. The latter are large (body up to 2 mm), have a long tail with fin-folds, numerous penetration gland cells that form 1–3 groups in the anterior part of the body, a well-developed Y-shaped excretory bladder with lateral diverticula in the stem and the arms and numerous flame cells (Stunkard, 1932; Rothschild, 1935; Wright, 1956).

Cable (1963) noted that excretory system of cercariae of Rhodometopa group was organized similarly to that in the adults. In the course of development of adult renicolids, the excretory bladder expands considerably and forms lateral diverticula, as it does in Rhodometopa cercariae. The number of flame cells also increases in the course of development, which is a characteristic of trematodes (Galaktionov and Dobrovolskij, 2003). On the basis of these observations, Cable (1963) suggested that Rhodometopa cercariae were more advanced than xiphidiocercariae and had certain traits of adult organization, particularly pronounced in the structure of their excretory system.

A series of transition forms from renicolid xiphidiocercariae to the cercariae of Rhodometopa group can be arranged. In cercariae of *R. buchanani* and *R. cerithidicola*, the site where the main collecting tube leaves the stem of the excretory bladder is shifted forwards; in *R. buchanani* it is located just before the bifurcation (Martin and Gregory, 1951; Martin, 1971). Cercariae of *C. caribbea* VII Cable, 1956, *C. caribbea* VIII, *C. caribbea* IX Cable, 1956 and *R. lari* not only lack the stylet, but also have a well-developed excretory bladder with lateral diverticula; the main collecting tube starts not from the stem but from the arms (Cable, 1956; Prevot and Bartoli, 1978). In drawings showing successive stages of embryogenesis in cercariae of *C. caribbea* VII (Cable, 1956; Plate 3, Fig. 16, p. 550) one can see that the site of the origin of main collecting tube, which in early embryos is located at the site of bifurcation of the excretory bladder, is shifted forwards together with the outgrown branches of the excretory bladder. This also seems to be the case during the ontogenesis of adults in renicolids with xiphidiocercariae, since their adults also have outgrown branches of the excretory bladder with numerous diverticula.

Another morphological character shared by the renicolid xiphidiocercariae and the Rhodometopa cercariae is the organization of surface structure in the oral and the ventral sucker. SEM studies of xiphidiocercariae of the ‘Parvicaudata’ group have revealed 1–2 rows of large spines in the suckers and 6 large uniciliated sensory papillae (2 anterior and 4 posterior) with a wide convex tegumental collars in the ventral sucker (Fig. 4D) (O'Dwyer *et al*., 2014; Denisova and Shchenkov, 2020; this study and our unpublished data). Rothschild (1935) noted a circle of spines and 6 large cuticular tubercles outside of them in the ventral sucker of a typical Rhodometopa cercaria *C. pythionike*. These ‘cuticular tubercles’ are arranged in the same manner as the sensory papillae in renicolid xiphidiocercariae and are, undoubtedly, sensory papillae, too.

The final evidence that Rhodometopa cercariae belong to renicolids came from the analysis of sequences of ITS2 rDNA of 2 typical larvae of Rhodometopa group: *C. pythionike* and *C. doricha* (Matos *et al*., 2019). This conclusion was supported by our analysis based on ITS2 rDNA sequences for a greater number of renicolid species (Fig. 8). To note, *C. pythionike* and *C. doricha* did not group with renicolids in the NCBI Blast analysis by Heneberg *et al*. (2016), which now seems to have been an error associated with the scarcity of the relevant sequences in the GenBank at the time of the analysis. In our phylogenetic tree both larvae of Rhodometopa group grouped with the species that belonged to clade II in *cox1*-based tree (Fig. 7). These 2 larvae clearly belong to different species. *Cercaria pythionike* is close to *R. sloanei*, but ASAP analysis convincingly shows that it is a distinct species.

It has been suggested that the formation of Rhodometopa cercariae in renicolids was associated with the colonization of plankton-eating fish as the second intermediate host and through them, of fish-eating seabirds such as alcids, penguins, petrels, pelicans, etc. (Wright, 1956; Odening, 1962; Cable, 1963; Prevot and Bartoli, 1978). This hypothesis is supported by the fact that *C. doricha* and *C. pythionike* group in the phylogenetic tree together with *R. sloanei*, a parasite of several species of penguins and alcids (Matos *et al*., 2019, 2021).

Renicolidae belong to the superfamily Microphalloidea Ward, 1901 (suborder Xiphidiata) (Cribb *et al*., 2003; Olson *et al*., 2003; Pérez-Ponce de León and Hernández-Mena, 2019), whose cercariae possess the stylet. Its origin is thought to be associated with the involvement of arthropods as the second intermediate host into the life cycle of the ancestral microphalloideans (Cribb *et al*., 2003). The cercaria uses the stylet to penetrate the arthropod cuticle or arthrodial membranes. In renicolid cercariae the stylet is reduced to some degree or even absent, as in the larvae of Rhodometopa group and ‘transitional morphotypes’. The reduction of the stylet is associated with the transition to the use of organisms without rigid cuticular covers, such as molluscs and fish, as the second intermediate host. Only a few of the stylet-bearing renicolid cercariae penetrate polychaetes (Hechinger and Miura, 2014) and occasionally crabs (Robson and Williams, 1970) alongside with molluscs.

Metacercariae of species with xiphidiocercariae develop in invertebrates inhabiting nearshore areas, usually the intertidal zone. Therefore, the range of their definitive hosts is limited by the birds feeding on these invertebrates such as gulls, terns and sandpipers. Colonization of fish-eating seabirds became possible after renicolids began to use fish, especially planktonic fish, as the second intermediate host. This transition called for new adaptations to the infection of second intermediate host by cercariae, and finally resulted in the evolution of the larvae of Rhodometopa type.

Morphological changes of the cercariae were also accompanied by the changes in their behavioural strategies. Stylet-bearing renicolid larvae, as most xiphidiocercariae, demonstrate an active searching strategy. They are constantly moving, searching and infecting animals with low mobility (in case of renicolids, mostly molluscs) (Prokofiev and Galaktionov, 2009; Nikolaev *et al*., 2017). In contrast, renicolid cercariae of ‘transitional morphotype’ and of Rhodometopa group exhibit intermittent swimming, alternating periods of active swimming with passive floating in the water column (Combes *et al*., 1994). In the passive phase, the cercariae acquire a characteristic resting pose, bending the tail to enhance the ‘parachuting’ effect and slow down the sinking (Cable, 1956, 1963; Prevot and Bartoli, 1978). An enlarged tail and the development of fin-folds, characteristic of Rhodometopa cercariae, serve the same aim. This behaviour corresponds to the active waiting strategy, characteristic of the cercariae infecting actively moving hosts such as fish (Prokofiev and Galaktionov, 2009). *C*ercaria *buchanani* unite by the proximal portions of their tails forming aggregations (Martin and Gregory, 1951), which corresponds to the prey mimetism strategy (Combes *et al*., 1994; Prokofiev and Galaktionov, 2009). Some elements of this strategy also seem to be characteristic of large Rhodometopa cercariae, which might be taken by the fish for food objects, e.g. small pelagic polychaetes. The example of renicolids illustrates a high plasticity of the structure of cercariae, which limits the use of the cercarial morphotype as a character for the establishment of taxa of high taxonomical level.

## Conclusions

We showed that the use of morphological criteria alone is insufficient for a revision of the Renicolidae. Characters such as the location of testes and vitellaria appear to have been evolving in a convergent manner in different phylogenetic branches of these digeneans. Based on our molecular analyses, we outlined 3 main branches of renicolids for which molecular data are available. Although our data are incomplete, we can tentatively suggest that the first branch (clade I) is characterized by parasitism of adults in gulls (possibly also in sandpipers) and by the presence of the xiphidiocercaria stage in the life cycle. Renicolid species from the second branch (clade II) use sea birds, including gulls, as the definitive host, and their cercariae belong to the Rhodometopa group or to ‘transitional morphotype’. The third branch is represented for now by 1 species, *R. somateriae*, a typical parasite of sea ducks, with xiphidiocercaria in the life cycle.

In our opinion, it is premature to attempt a thorough taxonomic revision of the renicolids. This task would be meaningful after the accumulation of molecular data, especially on morphologically contrasting species, the elucidation of life cycles of a greater number of species and the determination of the range of their hosts. A detailed analysis of the morphological features of adults and cercariae is also necessary.

## Data Availability

Data available on request from the authors.

## Supplementary material

For supplementary material accompanying this paper visit https://doi.org/10.1017/S0031182022001500. click here to view supplementary material

## References

[ref1] Anderson CM, Gilchrist HG, Ronconi RA, Shlepr KR, Clark DE, Fifield DA, Robertson GJ and Mallory ML (2020) Both short and long distance migrants use energy-minimizing migration strategies in North American herring gulls. Movement Ecology 8, 26. 32549986 10.1186/s40462-020-00207-9 PMC7294659

[ref2] Belopol'skaya MM (1952) Parasite fauna of marine waterfowl. Uchenie Zapiski Leningradskogo Universiteta *(*Scientific Reports of Leningrad State University*)* 141, ser. Biology 28, 127–180.

[ref3] Blakeslee AMH and Byers JE (2008) Using parasites to inform ecological history: comparisions among three congeneric marine snails. Ecology 89, 1068–1078. 18481531 10.1890/07-0832.1

[ref4] Blakeslee AMH and Fowler AE (2012) Aquatic introductions and genetic founder effects: how do parasites compare to hosts? In Caliskan M (ed.), Analysis of Genetic Variation in Animals. London, UK: IntechOpen, pp. 315–336. Available at doi: 10.5772/34089.

[ref5] Blakeslee AMH, Byers JE and Lesser P (2008) Solving cryptogenic histories using host and parasite molecular genetics: the resolution of *Littorina littorea*'s North American origin. Molecular Ecology 17, 3684–3696. 18643882 10.1111/j.1365-294X.2008.03865.x

[ref6] Blakeslee AMH, Miller AW, Ruiz GM, Johannesson K, André C and Panova M (2021) Population structure and phylogeography of two North Atlantic *Littorina* species with contrasting larval development. Marine Biology 168, 117.

[ref7] Blasco-Costa I and Poulin R (2017) Parasite life-cycle studies: a plea to resurrect an old parasitological tradition. Journal of Helminthology 91, 647–656. 28166844 10.1017/S0022149X16000924

[ref8] Blasco-Costa I, Cutmore S, Miller TL and Nolan MJ (2016) Molecular approaches to trematode systematics: ‘best practice’ and implications for future study. Systematic Parasitology 93, 295–306. 26898592 10.1007/s11230-016-9631-2

[ref9] Bowles J, Blair D and McManus DP (1992) Genetic variants within the genus *Echinococcus* identified by mitochondrial DNA sequencing. Molecular and Biochemical Parasitology 54, 165–173. 1435857 10.1016/0166-6851(92)90109-w

[ref10] Byrd EE and Heard RW (1970) Two new kidney flukes of the genus *Renicola* Cohn, 1904 from the Clapper Rail, *Rallus longirostris* subspp. The Journal of Parasitology 56, 493–497.

[ref11] Byrd EE and Kellogg FE (1972) *Renicola hayesannieae*, a new kidney fluke (Digenea: Renicolidae) from the wild turkey, *Meleagris gallopavo silvestris* Vieillot, from Mississippi. The Journal of Parasitology 58, 99–103. 5012530

[ref12] Caballero CE (1953) Helminths from the Republic Panama. VI. A new trematode of the family Renicolidae Dollfus, 1939. In Dayal J and Singh KS (eds), Thapar Commemoration Volume 1953: A Collection of Articles Pres. to Prof. GS Thapar on his 60th Birthday. Lucknow, India: University of Lucknow, pp. 25–30.

[ref13] Cable, RM (1956) Marine cercariae of Puerto Rico. In Miner RW (ed.), Scientific Survey of Porto Rico and the Virgin Islands. New York: New York Academy of Sciences, pp. 491–577.

[ref14] Cable RM (1963) Marine cercariae from Curaçao and Jamaica. Zeitschrift für Parasitenkunde 23, 429–469. 14111300 10.1007/BF00259930

[ref15] Campbell JG and Sloan J (1943) A possible new species of trematode parasitic in the kidneys of the king penguin (*Aptenodytes longirostris*). The Veterinary Journal 99, 291–294.

[ref16] Cannon LRG (1978) Marine cercariae from the gastropod *Cerithium moniliferum* Kiener at Heron Island, Great Barrier Reef. Proceedings of the Royal Society of Queensland 89, 45–57.

[ref17] Cannon LRG (1979) Ecological observations on *Cerithium moniliferum* Kiener (Gastropoda: Cerithiidae) and its trematode parasites at Heron Island, Great Barrier Reef. Australian Journal of Marine and Freshwater Research 30, 365–374.

[ref18] Clairbaux M, Fort J, Mathewson P, Porter W, Strøm H and Grémillet D (2019) Climate change could overturn bird migration: transarctic flights and high-latitude residency in a sea ice free Arctic. Scientific Reports 9, 17767. 31780706 10.1038/s41598-019-54228-5 PMC6883031

[ref19] Combes C, Fournier A, Moné H and Théron A (1994) Behaviours in trematode cercariae that enhance parasite transmission: patterns and processes. Parasitology 109(Suppl.), S3–S13. 7854849 10.1017/s0031182000085048

[ref20] Cribb TH, Bray RA, Olson PD and Littlewood DTJ (2003) Life cycle evolution in the Digenea: a new perspective from phylogeny. Advances in Parasitology 54, 197–254. 14711086 10.1016/s0065-308x(03)54004-0

[ref21] Davis SE, Maftei M and Mallory ML (2016) Migratory connectivity at high latitudes: Sabine's Gulls (*Xema sabini*) from a colony in the Canadian High Arctic migrate to different oceans. PLoS ONE 11, e0166043. 27973614 10.1371/journal.pone.0166043 PMC5156335

[ref22] Denisova SA and Shchenkov SV (2020) New data on the nervous system of *Cercaria parvicaudata* Stunkard & Shaw, 1931 (Trematoda: Renicolidae): revisiting old hypotheses. Journal of Helminthology 94, e52, 1–11. 10.1017/S0022149X1900035X 31084661

[ref23] Dyachenko V, Beck E, Pantchev N and Bauer C (2008) Cost-effective method of DNA extraction from taeniid eggs. Parasitology Research 102, 811–813. 18172686 10.1007/s00436-007-0855-6

[ref24] Ezhov AV, Gavrilo MV, Krasnov YV, Bråthen VS, Moe B, Baranskaya AV and Strøm H (2021) Transpolar and bi-directional migration strategies of black-legged kittiwakes *Rissa tridactyla* from a colony in Novaya Zemlya, Barents Sea, Russia. Marine Ecology Progress Series 676, 189–203.

[ref25] Flores K, López Z, Levicoy D, Muñoz-Ramírez CP, González-Wevar C, Oliva ME and Cárdenas L (2019) Identification assisted by molecular markers of larval parasites in two limpet species (Patellogastropoda: *Nacella*) inhabiting Antarctic and Magellan coastal systems. Polar Biology 42, 1175–1182.

[ref26] Galaktionov KV and Dobrovolskij AA (2003) The Biology and Evolution of Trematodes. An Essay on the Biology, Morphology, Life Cycles, Transmissions, and Evolution of Digenetic Trematodes. Boston, Dordrecht & London: Kluwer Academic.

[ref27] Galaktionov KV and Skírnisson K (2000) Digeneans from intertidal molluscs of SW Iceland. Systematic Parasitology 47, 87–101. 10966216 10.1023/a:1006426117264

[ref28] Galaktionov KV, Solovyeva AI and Miroliubov A (2021) Elucidation of *Himasthla leptosoma* (Creplin, 1829) Dietz, 1909 (Digenea, Himasthlidae) life cycle with insights into species composition of the north Atlantic *Himasthla* associated with periwinkles *Littorina* spp. Parasitology Research 120, 1649–1668. 33712931 10.1007/s00436-021-07117-8

[ref29] Gibson DI (2008) Family Renicolidae Dollfus, 1939. In Gibson DI, Bray RA and Jones A (eds), Keys to Trematoda, vol. 3. London, UK: CABI publishing, pp. 591–594.

[ref30] Gonchar A and Galaktionov KV (2021) It's marine: distinguishing a new species of *Catatropis* (Digenea: Notocotylidae) from its freshwater twin. Parasitology 148, 74–83. 32958097 10.1017/S0031182020001808 PMC11010198

[ref31] Gonchar A and Galaktionov KV (2022) The Pacific *Notocotylus atlanticus* (Digenea: Notocotylidae). Parasitology International 88, 102559. 35151846 10.1016/j.parint.2022.102559

[ref32] Granovitch AI and Johannesson K (2000) Digenetic trematodes in four species of *Littorina* from the West coast of Sweden. Ophelia 53, 55–65.

[ref33] Hallgrimsson GT, Gunnarsson HV, Torfason O, Buijs R-J and Camphuysen CJ (2012) Migration pattern of Icelandic lesser black-backed gulls *Larus fuscus graellsii*: indications of a leap-frog system. Journal of Ornithology 153, 603–609.

[ref34] Hechinger R (2007) Annotated key to the trematode species infecting *Batillaria attramentaria* (Prosobranchia: Batillariidae) as first intermediate host. Parasitology International 56, 287–296. 17644398 10.1016/j.parint.2007.06.004

[ref35] Hechinger R (2019) Guide to the trematodes (Platyhelminthes) that infect the California horn snail (*Cerithideopsis californica*: Potamididae: Gastropoda) as first intermediate host. Zootaxa 4711, 459–494. 10.11646/zootaxa.4711.3.3 32230486

[ref36] Hechinger RF and Miura O (2014) Two ‘new’ renicolid trematodes (Trematoda: Digenea: Renicolidae) from the California horn snail, *Cerithidea californica* (Haldeman, 1840) (Gastropoda: Potamididae). Zootaxa 3784, 559–574. 24872073 10.11646/zootaxa.3784.5.5

[ref37] Helberg M, Systad GH, Birkeland I, Lorentzen NH and Bustnes JO (2009) Migration patterns of adult and juvenile lesser black-backed gulls *Larus fuscus* from northern Norway. Ardea 97, 281–286.

[ref38] Heneberg P, Sitko J, Bizos J and Horne EC (2016) Central European parasitic flatworms of the family Renicolidae Dollfus, 1939 (Trematoda: Plagiorchiida): molecular and comparative morphological analysis rejects the synonymization of *Renicola pinguis* complex suggested by Odening. Parasitology 143, 1592–1604. 27356772 10.1017/S0031182016000895

[ref39] Hill WCO (1952) Report of the Society's Prosector for the year 1951. Proceedings of the Zoological Society of London 122, 515–553.

[ref40] Hill WCO (1954) Report of the Society's Prosector for the year 1953. Proceedings of the Zoological Society of London 124, 303–311.

[ref41] Holliman RB (1961) Larval trematodes from the Apalachee Bay area, Florida, with a checklist of known marine cercariae arranged in a key to their superfamilies. Tulane Studies in Zoology 9, 2–74.

[ref42] Huston DC, Cutmore SC and Cribb TH (2018) Molecular systematics of the digenean community parasitising the cerithiid gastropod *Clypeomorus batillariaeformis* Habe & Kusage on the Great Barrier Reef. Parasitology International 67, 722–735. 30053543 10.1016/j.parint.2018.07.008

[ref43] James BL (1968 *a*) The distribution and keys of species in the family Littorinidae and their digenean parasites, in the region of Dale, Pembrokeshire. Field Studies 2, 615–650.

[ref44] James BL (1968 *b*) The occurrence of larval Digenea in ten species of intertidal prosobranch molluscs in Cardigan Bay. Journal of Natural History 2, 329–343.

[ref45] James BL (1969) The Digenea of the intertidal prosobranch, *Littorina saxatilis* (Olivi). Zeitschrift für Zoologie, Systematik und Evolutionforschung 7, 273–316.

[ref46] Jenkins TL, Castilho R and Stevens JR (2018) Meta-analysis of northeast Atlantic marine taxa shows contrasting phylogeographic patterns following post-LGM expansions. PeerJ 6, e5684. 30280047 10.7717/peerj.5684 PMC6166638

[ref47] Jerdy H, Baldassin P, Werneck MR, Bianchi M, Ribeiro RB and Carvalho ECQ (2016) First report of kidney lesions due to *Renicola* sp. (Digenea: Trematoda) in free-living Magellanic penguins (*Spheniscus magellanicus* Forster, 1781) found on the coast of Brazil. The Journal of Parasitology 102, 650–652. 27552082 10.1645/16-29

[ref48] Kearse M, Moir R, Wilson A, Stones-Havas S, Cheung M, Sturrock S, Buxton S, Cooper A, Markowitz S, Duran C, Thierer T, Ashton B, Meintjes P and Drummond A (2012) Geneious basic: an integrated and extendable desktop software platform for the organization and analysis of sequence data. Bioinformatics 28, 1647–1649. 22543367 10.1093/bioinformatics/bts199 PMC3371832

[ref49] Kharoo VK (2013) A review of the history and classification of the family Renicolidae Dollfus, 1939 (Trematoda: Digenea). Indian Journal of Fundamental and Applied Life Sciences 3, 6–12.

[ref50] Klaassen RHG, Ens BJ, Shamoun-Baranes J, Exo K-M and Bairleind F (2012) Migration strategy of a flight generalist, the lesser black-backed gull *Larus fuscus*. Behavioral Ecology 23, 58–68.

[ref51] Kontrimavitschus VL and Bachmet'eva TL (1960) Helminth fauna of loons in the lover Lena. Trudy Gel'mintologicheskii Laboratoriia (Proceedings of the Helmintological Laboratory of the USSR Academy of Sciences) 10, 124–133.

[ref52] Kumar S, Stecher G, Li M, Knyaz C and Tamura K (2018) MEGA X: molecular evolutionary genetics analysis across computing platforms. Molecular Biology and Evolution 35, 1547–1549. 29722887 10.1093/molbev/msy096 PMC5967553

[ref53] Lauckner G (1980) Diseases of Mollusca: Gastropoda. In Kinne O (ed.), Diseases of Marine Animals, vol. 1. Chichester, New York, Brisbane, Toronto: John Wiley & Sons, pp. 311–424.

[ref54] Lauckner G (1983) Diseases of Mollusca: Bivalvia. In Kinne O (ed.), Diseases of Marine Animals, vol. 2. Hamburg: Biologische Anstalt Helgoland, pp. 477–961.

[ref55] Lauckner G (1987) Ecological effects of larval trematode infestation on littoral marine invertebrate populations. International Journal for Parasitology 47, 391–398.

[ref56] Lebour MV (1911) A review of the British marine cercariae. Parasitology 4, 416–456.

[ref57] Leigh JW and Bryant D (2015) PopART: full-feature software for haplotype network construction. Methods in Ecology and Evolution 6, 1110–1116.

[ref58] Leonov VA, Belogurov OI, Shagvaleeva NM and Bondarenko SK (1963) To trematode fauna of fish-eating birds of Kamchatka. In Leonov VA, Mamaev YuL and Oshmarin PG (eds), Paraziticheskie chervil domashnih I dikih szivotnih (Parasitic Worms of Domestic and Wild Animals). Vladivostok, USSR: Far East Department of the Siberian Branch of the USSR Academy of Sciences Press, pp. 130–195.

[ref59] Leung TLF, Donald KM, Keeney DB, Koehler AV, Peoples RC and Poulin R (2009) Trematode parasites of Otago Harbour (New Zealand) soft-sediment intertidal ecosystems: life cycles, ecological roles and DNA barcodes. New Zealand Journal of Marine and Freshwater Research 43, 857–865.

[ref60] Litvaitis MK and Rohde K (1999) A molecular test of platyhelminth phylogeny: inferences from partial 28S rDNA sequences. Invertebrate Biology 18, 42–56.

[ref61] Maggs CA, Castilho R, Foltz D, Henzler C, Jolly MT, Kelly J, Olsen J, Perez KE, Stam W, Vainola R, Viard F and Wares J (2008) Evaluating signatures of glacial refugia for North Atlantic benthic marine taxa. Ecology 89, S108–S122. 19097488 10.1890/08-0257.1

[ref62] Mahdy OA and Shaheed IB (2001) Histopathological study on the effect of *Renicola heroni* on the kidneys of giant heron *Ardea goliath*. Helminthologia 38, 81–83.

[ref63] Martin WE (1971) Larval stages of renicolid trematodes. Transactions of the American Microscopical Society 90, 188–194.

[ref64] Martin WE and Gregory VL (1951) *Cercaria buchanani* n. sp., an aggregating marine trematode. Transactions of the American Microscopic Society 70, 359–362.

[ref65] Martorelli SR, Fredensborg BL, Leung TLF and Poulin R (2008) Four trematode cercariae from the New Zealand intertidal snail *Zeacumantus subcarinatus* (Batillariidae). New Zealand Journal of Zoology 35, 73–84.

[ref66] Matos AMRN, Lavorente FLP, Lorenzetti E, Meira-Filho MRC, Nóbrega DF, Chryssafidis AL, Oliveira AG, Domit C and Bracarense APFRL (2019) Molecular identification and histological aspects of *Renicola sloanei* (Digenea: Renicolidae) in *Puffinus puffinus* (Aves: Procellariiformes): a first record. Revista Brasileira de Parasitologia Veterinária 28, 367–375. 31483030 10.1590/S1984-29612019025

[ref67] Matos AMRN, Meira-Filho MRC, Lorenzetti E, Lavorente FLP, Caldart ET, Bizari TG, Matos RLN, Domit C and Bracarense APFRL (2021) Renicolidae infection in Manx shearwater (*Puffinus puffinus*): is parasitism implicated on renal lesions? Parasitology Research 120, 1311–1320. 33594620 10.1007/s00436-020-06959-y

[ref68] Mouritsen KN and Elkjær CK (2020) Cost of interspecific competition between trematode colonies. Journal of Helminthology 94, e139, 1–6. 32238196 10.1017/S0022149X20000243

[ref69] Munyer PD and Holloway HL (1990) *Renicola williamsi* n.sp. (Trematoda; Digenea; Renicolidae) from the South Polar Skua, *Catharacta maccormiki*. Transactions of the American Microscopical Society 109, 98–102.

[ref70] Nadakal AM (1960) Type and source of pigments in certain species of larval trematodes. The Journal of Parasitology 46, 777–786. 13727319

[ref71] Newell CR (1986) The marine fauna and flora of the Isles of Scilly: some marine digeneans from invertebrate hosts. Journal of Natural History 20, 71–77.

[ref72] Newton I (2010) Bird Migration. London, UK: Collins.

[ref73] Nikolaev KE, Prokofiev VV, Levakin IA and Galaktionov KV (2017) How the position of mussels at the intertidal lagoon affects their infection with the larvae of parasitic flatworms (Trematoda: Digenea): A combined laboratory and field experimental study. Journal of Sea Research 128, 32–40.

[ref74] Nikolaev KE, Levakin IA and Galaktionov KV (2021) A month for the mission: using a sentinel approach to determine the transmission window of digenean cercariae in the subarctic White Sea. Journal of Helminthology 95, e50, 1–6. 34429183 10.1017/S0022149X21000456

[ref75] Odening K (1962) Neue Trematoden aus Vietnamesischen Vogeln des Berliner Tierparks (Mit einer Revision der Familie Renicolidae). Bijdragen tot de Dierkunde 32, 49–63.

[ref76] O'Dwyer K, Blasco-Costa I, Poulin R and Faltýnková A (2014) Four marine digenean parasites of *Austrolittorina* spp. (Gastropoda: Littorinidae) in New Zealand: morphological and molecular data. Systematic Parasitology 89, 133–152. 25204600 10.1007/s11230-014-9515-2

[ref77] O'Dwyer K, Faltýnková A, Georgieva S and Kostadinova A (2015) An integrative taxonomic investigation of the diversity of digenean parasites infecting the intertidal snail *Austrolittorina unifasciata* Gray, 1826 (Gastropoda: Littorinidae) in Australia. Parasitology Research 114, 2381–2397. 25866083 10.1007/s00436-015-4436-9

[ref78] Olson PD, Cribb TH, Tkach VV, Bray RA and Littlewood DTJ (2003) Phylogeny and classification of the Digenea (Platyhelminthes: Trematoda). International Journal for Parasitology 33, 733–755. 12814653 10.1016/s0020-7519(03)00049-3

[ref79] Palm HW, Waeschenbach A, Olson PD and Littlewood DTJ (2009) Molecular phylogeny and evolution of the Trypanorhyncha Diesing, 1863 (Platyhelminthes: Cestoda). Molecular Phylogenetics and Evolution 52, 351–367. 19489123 10.1016/j.ympev.2009.01.019

[ref80] Patitucci KF, Kudlai O and Tkach VV (2015) *Nephromonorcha varitestis* n. sp. (Digenea: Renicolidae) from the American White Pelican, *Pelecanus erythrorhynchos* in North Dakota, U.S.A. Comparative Parasitology 82, 254–261.

[ref81] Pérez-Ponce de León G and Hernández-Mena DI (2019) Testing the higher-level phylogenetic classification of Digenea (Platyhelminthes, Trematoda) based on nuclear rDNA sequences before entering the age of the ‘next-generation’ Tree of Life. Journal of Helminthology 93, 260–276. 30973318 10.1017/S0022149X19000191

[ref82] Prevot G and Bartoli P (1978) Le cycle de developpement de *Renicola lari* J. Timon-David, 1933 (Trematoda, Renicolidae). Annales de Parasitologie (Paris) 53, 561–575. 10.1051/parasite/1978536561 754615

[ref83] Prokofiev VV and Galaktionov KV (2009) Strategies of search behaviour in trematode cercariae. Proceedings of the Zoological Institute of the Russian Academy of Sciences 313, 308–318.

[ref84] Puillandre N, Brouillet S and Achaz G (2020) ASAP: assemble species by automatic partitioning. Molecular Ecology Resources 21, 609–620. 33058550 10.1111/1755-0998.13281

[ref85] Reid DG (1996) Systematics and Evolution of Littorina. London, UK: The Ray Society.

[ref86] Reid DG, Rumbak E and Thomas RH (1996) DNA, morphology and fossils: phylogeny and evolutionary rates of the gastropod genus *Littorina*. Philosophical Transactions of the Royal Society of London B 351, 877–895. 10.1098/rstb.1996.0082 8856807

[ref87] Reid DG, Dyal P and Williams ST (2012) A global molecular phylogeny of 147 periwinkle species (Gastropoda, Littorininae). Zoologica Scripta 41, 125–136.

[ref88] Richard J (1971) La chétotaxie des cercaries. Valeur systématique et phylétique. Mémoires du Muséum national d'histoire náturelle 67, 4–177.

[ref89] International Commission on Zoological Nomenclature (ICZN) (1999) International Code of Zoological Nomenclature, 4th Edn. Ride WDL, Cogger HG, Dupuis C, Kraus O, Minelli A, Thompson FC and Tubbs PK (eds). London, UK: The International Trust for Zoological Nomenclature. Available at https://www.iczn.org/the-code/the-code-online/.

[ref90] Riley T and Owen RW (1972) *Renicola glacialis* sp. nov. a new trematode from the North Sea fulmar *Fulmarus glacialis* (L.), with observations on its pathology. Journal of Helminthology 46, 63–72. 5038425

[ref91] Robson EM and Williams IC (1970) Relationships of some species of Digenea with the marine prosobranch *Littorina littorea* (L.) I. The occurrence of larval digenea in *L. littorea* on the North Yorkshire Coast. Journal of Helminthology 44, 163–168.

[ref92] Ronquist F, Teslenko M, van der Mark P, Ayres DL, Darling A, Höhna S, Larget B, Liu L, Suchard MA and Huelsenbeck JP (2012) MrBayes 3.2: Efficient Bayesian Phylogenetic inference and model choice across a large model space. Systematic Biology 61, 539–542. doi: 10.1093/sysbio/sys029 22357727 PMC3329765

[ref93] Rothschild M (1935) The trematode parasites of *Turritella communis* Lmk. from Plymouth and Naples. Parasitology 27, 152–157.

[ref94] Rozas J, Ferrer-Mata A, Sánchez-DelBarrio JC, Guirao-Rico S, Librado P, Ramos-Onsins SE and Sánchez-Gracia A (2017) DnaSP 6: DNA sequence polymorphism analysis of large datasets. Molecular Biology and Evolution 34, 3299–3302. 29029172 10.1093/molbev/msx248

[ref95] Rubio-Godoy M, Pérez-Ponce de León G, Mendoza-Garfias B, Carmona-Isunzaf MC, la Moral N and Drummondt H (2011) Helminth parasites of the blue-footed booby on Isla Isabel, Mexico. The Journal of Parasitology 97, 636–641. 21506815 10.1645/GE-2675.1

[ref96] Rybakov AV (1983) Fauna i ecologia trematode massovih vidov molluscov severo-zapadnoi chasti Yaposkogo moria (Fauna and ecology of trematodes of the mass species of molluscs of the western part of the Sea of Japan) (PhD thesis). Vladivostok, Leningrad, USSR. Manuscript available at https://www.dissercat.com/content/fauna-i-ekologiya-trematod-massovykh-vidov-mollyuskov-severo-zapadnoi-chasti-yaponskogo-mory (in Russian).

[ref97] Sannia A and James BL (1977) The digenea in marine molluscs from Eyjafjördur, North Iceland. Ophelia 16, 97–109.

[ref98] Skírnisson K and Galaktionov KV (2002) Life cycles and transmission patterns of seabird digeneans in SW Iceland. Sarsia 87, 144–151.

[ref99] Skírnisson K, Guðmundsdóttir B, Andrésdóttir V and Galaktionov KV (2002–2003) ITS1 nuclear rDNA sequences used to clear the life cycle of the morphologically different larvae and adult renicolid (Renicola, Digenea) parasites found in Iceland. Bulletin of the Scandinavian Society for Parasitology 12–13, 50.

[ref100] Sonsthagen SA, Chesser RT, Bell DA and Dove CJ (2012) Hybridization among Arctic white-headed gulls (*Larus* spp.) obscures the genetic legacy of the Pleistocene. Ecology and Evolution 2, 1278–1295. 22833800 10.1002/ece3.240 PMC3402200

[ref101] Stunkard HW (1932) Some larval trematodes from the coast in the region of Roscoff, Finistere. Parasitology 24, 321–343.

[ref102] Stunkard HW (1950) Further observations on *Cercaria parvicaudata* Stunkard and Shaw, 1931. Biological Bulletin of the Marine Laboratory, Woods Hole 99, 136–142. 10.2307/1538757 14772249

[ref103] Stunkard HW (1964) Studies on the trematode genus *Renicola*: observations on the life-history, specificity, and systematic position. Biological Bulletin of the Marine Laboratory, Woods Hole 126, 468–489.

[ref104] Stunkard HW (1971) Renicolid trematodes (Digenea) from the renal tubules of birds. Annales de Parasitologie (Paris) 46, 109–118. 10.1051/parasite/1971461109 4935277

[ref105] Stunkard HW and Shaw CR (1931) The effect of dilution of sea water on the activity and longevity of certain marine cercariae, with description of two new species. Biological Bulletin of the Marine Laboratory, Woods Hole 61, 242–271.

[ref106] Stunkard HW, Nigrelli RF and Gandal ChP (1958) The morphology of *Renicola philippinensis*, n. sp., a digenetic trematode from the pheasant-tailed Jacana, *Hydrophasianus chirurgus* (Scopoli). Zoologica: Scientific Contributions of the New York Zoological Society 43, 105–112.

[ref107] Sudarikov VE and Stenko RP (1984) Trematodes of the family Renicolidae. In Sonin MD (ed.), Helminths of Farming and Hunting Animals. Moscow, USSR: Nauka, pp. 34–89 (in Russian).

[ref108] Tamura K, Stecher G, Peterson D, Filipski A and Kumar S (2013) MEGA6: molecular evolutionary genetics analysis version 6.0. Molecular Biology and Evolution 30, 2725–2729. 24132122 10.1093/molbev/mst197 PMC3840312

[ref109] Thieltges DW (2006) Effect of infection by metacercarial trematode *Renicola roscovita* on growth in intertidal blue mussel *Mytilus edulis*. Marine Ecology Progress Series 319, 129–134.

[ref110] Thieltges DW and Rick J (2006) Effect of temperature on emergence, survival and infectivity of cercariae of the marine trematode *Renicola roscovita* (Digenea: Renicolidae). Diseases of Aquatic Organisms 73, 63–68. 17240753 10.3354/dao073063

[ref111] Tkach VV, Pawlowski J, Mariaux J and Swiderski Z (2001) Molecular phylogeny of the suborder Plagiorchiata and its position in the system of Digenea. In Littlewood DTJ and Bray RA (eds), Interrelationships of Platyhelminthes. London, UK: Taylor & Francis, pp. 186–193.

[ref112] Tsimbaljuk AK, Kulikov VV, Ardasheva NV and Tsimbaljuk EM (1978) Helminths of invertebrates from the intertidal zone of the Iturup island. In Kusakin OG (ed.), Szivotniy i rastitel'niy mir shelfovih zon Kurilskih ostrovov (Fauna and Vegetation of the Shelf of the Kuril Islands). Moscow, USSR: Nauka, pp. 69–126.

[ref113] Wares JP and Cunningham CW (2001) Phylogeography and historical ecology of the North Atlantic intertidal. Evolution 55, 2455–2469. 11831661 10.1111/j.0014-3820.2001.tb00760.x

[ref114] Werding B (1969) Morphologie, Entwicklung und Ökologie digener Trematoden-Larven der Strandschnecke *Littorina littorea*. Marine Biology 3, 306–333.

[ref115] Winnepenninckx B, Backeljau T and De Wachter R (1993) Extraction of high molecular weight DNA from molluscs. Trends in Genetics 9, 407. 8122306 10.1016/0168-9525(93)90102-n

[ref116] Wright CA (1953) Probable relationship between the Rhodometopa group of cercariae and the trematode genus *Renicola* Cohn. Nature 171, 1072–1073. 10.1038/1711072b0 13063535

[ref117] Wright CA (1954) Trematodes of the genus *Renicola* from birds in British zoos, with descriptions of two new species. Proceedings of the Zoological Society of London 124, 51–61.

[ref118] Wright CA (1956) Studies on the life history and ecology of the trematode genus *Renicola* Cohn, 1904. Proceedings of the Zoological Society of London 126, 1–49.

[ref119] Wright CA (1957) Two kidney flukes from Sudanese birds with a description of a new species. Journal of Helminthology 31, 229–238. 13491828 10.1017/s0022149x00004478

[ref120] Yamaguti S (1939) Studies on the helminth fauna of Japan. Part 25. Trematodes of birds, IV. Japanese Journal of Zoology 8, 129–210.

