# Supplementary material for: Overview of renicolid digeneans (Digenea, Renicolidae) from marine gulls of northern Holarctic with remarks on their species statuses, phylogeny and phylogeography
Source: Parasitology. 2022 Nov 2;150(1):55–77. doi: 10.1017/S0031182022001500 (PMC10090622; doi:10.1017/S0031182022001500)
Supplement: Supplementary file 1 [file S0031182022001500sup.zip › S0031182022001500sup002.docx]

Table S1a. Mean genetic divergence between *Renicola*, *Nephromonorcha* and *Deretrema* species estimated as p-distance for the D1-D3 fragment of 28S rRNA gene. The number of base differences per site from averaging over all sequence pairs between groups (above the diagonal) and standard error estimates (below the diagonal) are shown. The name of *C. littorinae saxatilis* VIII group is shortened as *C. lit. sax.* VIII.

| Groups | Aus1 | Aus2 | *Renicola* sp. | *Deretrema* | *Nephromonorcha* | *R. parvicaudatus* | *C. lit. sax.*VIII | *R. keimahuri* | *R. somateria* |
| --- | --- | --- | --- | --- | --- | --- | --- | --- | --- |
| Aus1 |  | 0.032 | 0.106 | 0.215 | 0.144 | 0.026 | 0.030 | 0.161 | 0.149 |
| Aus2 | 0.005 |  | 0.108 | 0.214 | 0.146 | 0.031 | 0.015 | 0.166 | 0.154 |
| *Renicola sp* | 0.009 | 0.009 |  | 0.214 | 0.139 | 0.102 | 0.105 | 0.165 | 0.146 |
| *Deretrema* | 0.012 | 0.012 | 0.012 |  | 0,207 | 0.219 | 0.213 | 0.220 | 0.214 |
| *Nephromonorcha* | 0.011 | 0.011 | 0.010 | 0.012 |  | 0.146 | 0.142 | 0.109 | 0.129 |
| *R. parvicaudatus* | 0.005 | 0.005 | 0.008 | 0.012 | 0.011 |  | 0.028 | 0.162 | 0.150 |
| *C. lit. sax.* VIII | 0.005 | 0.003 | 0.008 | 0.012 | 0.010 | 0.005 |  | 0.158 | 0.147 |
| *R. keimahuri* | 0.011 | 0.011 | 0.011 | 0.012 | 0.010 | 0.011 | 0.011 |  | 0.144 |
| *R. somateria* | 0.011 | 0.011 | 0.010 | 0.012 | 0.010 | 0.011 | 0.011 | 0.011 |  |

Groups include the following isolates: Aus1 (KP903411.1 *Renicola* sp. 1 Aus isolate AUAM2, KP903412.1 *Renicola* sp. 1 Aus isolate AUSD3), Aus2 (KP903413.1 *Renicola* sp. 2 Aus isolate AUAMP1), *Renicola* sp. (MH257770.1 *Renicola* sp., AY116871.1 *Renicola* sp. VT-2002), *Deretrema* (AY222273.1 *Deretrema nahaense*), Nephromonorcha (KP710186.1 *N. varitestis* isolate 2, KP710187.1 *Nephromonorcha varitestis* isolate 3), *R. parvicaudatus* (1siOP, 32saxIC, 27litHR, 58siOP), *C. littorinae saxatilis* VIII (7saxIP, 26saxBP, 57obtBP), *R. keimahuri* (8OmR), *R. somateria* (10 nIR).

Table S1b. Mean genetic divergence between *Renicola*, *Nephromonorcha* and *Deretrema* species estimated as p-distance for the D1-D3 fragment of 28S rRNA gene. Isolates 1siOP and 58siOP were separated and analysed as independent groups. The number of base differences per site from averaging over all sequence pairs between groups (above the diagonal) and standard error estimates(below the diagonal) are shown. The name of *C. littorinae saxatilis* VIII group is shortened as *C. lit. sax.* VIII.

| Groups | Aus1 | Aus2 | *Nephromonorcha* | 58siop | *C. lit. sax.VIII* | *R. keimahuri* | *R. parvicaudatus* | 1siop | *R. somateria* |
| --- | --- | --- | --- | --- | --- | --- | --- | --- | --- |
| Aus1 |  | 0.032 | 0.144 | 0.028 | 0.030 | 0.161 | 0.025 | 0.027 | 0.149 |
| Aus2 | 0.005 |  | 0.146 | 0.033 | 0.015 | 0.166 | 0.030 | 0.032 | 0.154 |
| *Nephromonorcha* | 0.011 | 0.011 |  | 0.147 | 0.142 | 0.109 | 0.146 | 0.146 | 0.129 |
| 58siop | 0.005 | 0.005 | 0.011 |  | 0.030 | 0.162 | 0.004 | 0.004 | 0.151 |
| *C. lit. sax.* VIII | 0.005 | 0.003 | 0.011 | 0.005 |  | 0.158 | 0.027 | 0.029 | 0.147 |
| *R. keimahuri* | 0.011 | 0.011 | 0.009 | 0.011 | 0.011 |  | 0.162 | 0.161 | 0.144 |
| *R. parvicaudatus* | 0.005 | 0.005 | 0.011 | 0.002 | 0.005 | 0.011 |  | 0.003 | 0.150 |
| 1siop | 0.005 | 0.005 | 0.011 | 0.002 | 0.005 | 0.011 | 0.002 |  | 0.151 |
| *R. somateria* | 0.010 | 0.011 | 0.010 | 0.010 | 0.010 | 0.010 | 0.010 | 0.010 |  |

Groups contain the same isolates as in table S1a and two additional ones: 1siOP and 58siOP isolates represent groups of the same names.

| Groups | *R. parvicaudatus* | *C. lit. sax.*VIII | Aus2 | Aus1 | *Renicola* sp | *Deretrema* | *Nephromonorcha* | *R. keimahuri* | *R. somateria* |
| --- | --- | --- | --- | --- | --- | --- | --- | --- | --- |
| *R.parvicaudatus* |  | 0.026 | 0.031 | 0.031 | 0.065 | 0.163 | 0.073 | 0.107 | 0.085 |
| *C. lit. sax.* VIII | 0.008 |  | 0.013 | 0.010 | 0.055 | 0.154 | 0.061 | 0.092 | 0.067 |
| Aus2 | 0.009 | 0.006 |  | 0.009 | 0.061 | 0.148 | 0.067 | 0.092 | 0.067 |
| Aus1 | 0.008 | 0.004 | 0.004 |  | 0.058 | 0.151 | 0.064 | 0.092 | 0.067 |
| *Renicola* sp | 0.012 | 0.012 | 0.013 | 0.012 |  | 0.147 | 0.064 | 0.107 | 0.061 |
| *Deretrema* | 0.020 | 0.020 | 0.020 | 0.019 | 0.019 |  | 0.133 | 0.145 | 0.130 |
| *Nephromonorcha* | 0.014 | 0.013 | 0.014 | 0.013 | 0.013 | 0.019 |  | 0.052 | 0.058 |
| *R. keimahuri* | 0.017 | 0.016 | 0.016 | 0.015 | 0.017 | 0.019 | 0.013 |  | 0.080 |
| *R. somateria* | 0.015 | 0.014 | 0.014 | 0.013 | 0.013 | 0.019 | 0.013 | 0.015 |  |

Table S1c. Mean genetic divergence between *Renicola*, *Nephromonorcha* and *Deretrema* species estimated as p-distance for the short fragment of *28S rRNA* gene. The number of base differences per site from averaging over all sequence pairs between groups(above the diagonal) and standard error estimates(below the diagonal) are shown. The name of *C. littorinae saxatilis* VIII group is shortened as *C. lit. sax.* VIII.

Groups contain the same isolates as in table S1a with one exception: *R. parvicaudatus* group also contains isolate AF023113.1 *Renicola roscovita.*

Table S1d. Intragroup mean genetic divergence in *Renicola* and *Nephromonorcha* species estimated as p-distance for the D1-D3 fragment of 28S rRNA gene. Groups contain the same isolates as in table S1a.

| Group | d | SD |
| --- | --- | --- |
| Aus1 | 0 | 0 |
| *Renicola* sp. | 0.049 | 0.007 |
| *Nephromonorcha* | 0 | 0 |
| *R. parvicaudatus* | 0.003 | 0.001 |
| *C. littorinae saxatilis* VIII | 0.003 | 0.001 |

Table S1e. Intragroup mean genetic divergence in *Renicola* and *Nephromonorcha* species estimated as p-distance for the short fragment of 28S rRNA gene. Groups contain the same isolates as in table S1c.

| Group | d | SD |
| --- | --- | --- |
| *R. parvicaudatus* | 0.011 | 0.004 |
| *C. littorinae saxatilis* VIII | 0.002 | 0.002 |
| Aus1 | 0.018 | 0.007 |
| *Renicola* sp. | 0.018 | 0.008 |
| *Nephromonorcha* | 0.000 | 0.000 |

Table S2a. Mean genetic divergence between *Renicola*, *Paragonimus* and *Microphallidae* species estimated as p-distance for the short fragment of *cox1* mitochondrial gene. The number of base differences per site from averaging over all sequence pairs between groups (above the diagonal) and standard error estimates (below the diagonal) are shown. The name of *C. littorinae saxatilis* VIII group is shortened as *C. lit. sax.* VIII.

| Groups | *R. parvicaudata* | *R. somateria* | *C. lit.sax* VIII | *R. keimahuri* | *Paragonimus* | *Renicola TLFL* | *Microphallidae* | *Renicola* sp.  *"polychaetophila"* | *Renicola* sp.  *"martini"* | Aus2 | Aus1 | *R. sternae* | *R. lari* |
| --- | --- | --- | --- | --- | --- | --- | --- | --- | --- | --- | --- | --- | --- |
| *R. parvicaudata* |  | 0.248 | 0.106 | 0.248 | 0.327 | 0.148 | 0.314 | 0.179 | 0.140 | 0.097 | 0.094 | 0.261 | 0.226 |
| *R. somateria* | 0.024 |  | 0.248 | 0.256 | 0.281 | 0.220 | 0.307 | 0.230 | 0.224 | 0.220 | 0.244 | 0.291 | 0.268 |
| *C. lit.sax.* VIII | 0.016 | 0.023 |  | 0.253 | 0.307 | 0.144 | 0.318 | 0.166 | 0.142 | 0.082 | 0.120 | 0.255 | 0.232 |
| *R. keimahuri* | 0.025 | 0.025 | 0.024 |  | 0.313 | 0.259 | 0.297 | 0.244 | 0.224 | 0.238 | 0.257 | 0.121 | 0.125 |
| *Paragonimus* | 0.027 | 0.026 | 0.026 | 0.026 |  | 0.293 | 0.332 | 0.304 | 0.284 | 0.300 | 0.312 | 0.316 | 0.284 |
| *Renicola TLFL* | 0.020 | 0.022 | 0.019 | 0.025 | 0.026 |  | 0.314 | 0.148 | 0.119 | 0.158 | 0.169 | 0.253 | 0.244 |
| *Microphallidae* | 0.024 | 0.026 | 0.025 | 0.025 | 0.027 | 0.026 |  | 0.334 | 0.326 | 0.316 | 0.318 | 0.323 | 0.284 |
| *Renicola* sp.  *"polychaetophila"* | 0.021 | 0.023 | 0.021 | 0.025 | 0.026 | 0.020 | 0.025 |  | 0.112 | 0.145 | 0.188 | 0.268 | 0.249 |
| *Renicola* sp.  *"martini"* | 0.019 | 0.022 | 0.019 | 0.024 | 0.025 | 0.018 | 0.026 | 0.018 |  | 0.133 | 0.142 | 0.252 | 0.227 |
| Aus2 | 0.016 | 0.023 | 0.014 | 0.024 | 0.026 | 0.020 | 0.025 | 0.020 | 0.018 |  | 0.105 | 0.268 | 0.230 |
| Aus1 | 0.016 | 0.024 | 0.018 | 0.025 | 0.026 | 0.021 | 0.026 | 0.022 | 0.020 | 0.017 |  | 0.264 | 0.244 |
| *R. sternae* | 0.025 | 0.025 | 0.024 | 0.018 | 0.026 | 0.025 | 0.025 | 0.025 | 0.025 | 0.025 | 0.025 |  | 0.115 |
| *R. lari* | 0.023 | 0.026 | 0.023 | 0.018 | 0.025 | 0.024 | 0.024 | 0.025 | 0.024 | 0.023 | 0.024 | 0.018 |  |

Groups include following isolates: Aus1 (KP903421.1 *Renicola* sp. 1 Aus isolate AUAM2, KP903422.1 *Renicola* sp. 1 Aus isolate AUSD3), Aus2 (KJ868205.1 *Renicola* sp. NZ isolate AAPA2, KP903423.1 *Renicola* sp. 2 Aus isolate AUAMP1), *R. parvicaudatus* (1siOP, 4ImR, 13saxWSP,14obtWSP, 31litWSR, 32saxIC, 27litHR, 58siOP, EUCPESBJE1, NACPBOOTH2, 11 Ersh, 12 Ersh), *C. lit. sax.* VIII (7saxIP, 26saxBP, 41saxBP, 42saxBP, 43saxBP, 57obtBP), *Renicola* TLFL (FJ765490.1 *Renicola* sp. TLFL-2009 isolate PNI01, FJ765491.1 *Renicola* sp. TLFL-2009 isolate Z,FJ765492.1 *Renicola* sp. TLFL-2009 isolate Otk, FJ765493.1 *Renicola* sp. TLFL-2009 isolate DB01), *Renicola* sp. *“martini”* (KF512553.1 *Renicola* sp. *martini* isolate 17 KF512554.1 *Renicola* sp. martini isolate 22), *Renicola* sp*. “polychaetophila”* (KF512551.1 *Renicola* sp. *polychaetophila* isolate 21, KF512552.1 *Renicola* sp. polychaetophila isolate 16), *R. sternae* (KU563722.1 *Renicola sternae* voucher 3LF-1159 KU563723.1 *Renicola sternae* voucher 3LF-1161), *R. lari* (KU563725.1 *Renicola lari* voucher 3LF-2443, KU563726.1 *Renicola lari* voucher 3LF-2444, KU563727.1 *Renicola lari* voucher 3LF-2459), *R. keimahuri* (8OmR), *R. somateria* (10nIR), *Paragonimus* (AB354223.1 *Paragonimus westermani)*, *Microphallidae* (FJ765510.1 *Microphallidae* sp. TLFL-2009).

Table S2b. Intragroup mean genetic divergence in *Renicola* species estimated as p-distance for the short fragment of 28S rRNA gene. Groups contain the same isolates as in table S2a.

| Groups | d | SD |
| --- | --- | --- |
| *R. parvicaudatus* | 0.006 | 0.002 |
| *C. littorinae saxatilis* VIII | 0.007 | 0.003 |
| *Renicola TLFL* | 0.002 | 0.002 |
| *Renicola* sp. *"polychaetophila"* | 0.010 | 0.005 |
| *Renicola* sp.*"martini"* | 0.000 | 0.000 |
| Aus2 | 0.035 | 0.010 |
| Aus1 | 0.003 | 0.003 |
| *R. sternae* | 0.000 | 0.000 |
| *R. lari* | 0.000 | 0.000 |

| Groups | *R. somateria* | *C. lit. sax.*VIII | *R. parvicaudatus* | *C. doricha* | *C. pythionike* | *R. sternae* | *R. pinguis* | *R. lari* | *R. sloanei* | *Microphallus* | *Fasciola* |
| --- | --- | --- | --- | --- | --- | --- | --- | --- | --- | --- | --- |
| *R. somateria* |  | 0.264 | 0.281 | 0.239 | 0.224 | 0.234 | 0.219 | 0.225 | 0.222 | 0.228 | 0.326 |
| *C. lit. sax.* VIII | 0.023 |  | 0.044 | 0.242 | 0.223 | 0.271 | 0.208 | 0.218 | 0.224 | 0.262 | 0.382 |
| *R. parvicaudatus* | 0.024 | 0.010 |  | 0.263 | 0.247 | 0.282 | 0.232 | 0.238 | 0.248 | 0.273 | 0.406 |
| *C. doricha* | 0.023 | 0.022 | 0.023 |  | 0.026 | 0.210 | 0.168 | 0.137 | 0.049 | 0.255 | 0.364 |
| *C. pythionike* | 0.023 | 0.022 | 0.022 | 0.009 |  | 0.172 | 0.127 | 0.101 | 0.014 | 0.228 | 0.352 |
| *R. sternae* | 0.023 | 0.024 | 0.023 | 0.022 | 0.021 |  | 0.122 | 0.132 | 0.169 | 0.273 | 0.395 |
| *R. pinguis* | 0.023 | 0.022 | 0.023 | 0.020 | 0.018 | 0.017 |  | 0.074 | 0.126 | 0.228 | 0.354 |
| *R. lari* | 0.023 | 0.022 | 0.022 | 0.019 | 0.017 | 0.018 | 0.014 |  | 0.103 | 0.247 | 0.372 |
| *R. sloanei* | 0.023 | 0.022 | 0.022 | 0.012 | 0.006 | 0.020 | 0.018 | 0.017 |  | 0.223 | 0.356 |
| *Microphallus* | 0.023 | 0.023 | 0.024 | 0.024 | 0.023 | 0.024 | 0.024 | 0.024 | 0.023 |  | 0.341 |
| *Fasciola* | 0.025 | 0.025 | 0.026 | 0.024 | 0.025 | 0.025 | 0.025 | 0.024 | 0.025 | 0.025 |  |

Table S4. Mean genetic divergence between *Renicolidae*, *Microphallus* and *Fasciola* species estimated as p-distance for the ITS2 fragment of *rRNA* gene. The number of base differences per site from averaging over all sequence pairs between groups (above the diagonal) and standard error estimates (below the diagonal) are shown. The name of *C. littorinae saxatilis* VIII group is shortened as *C. lit. sax.* VIII.

# Groups include following isolates: *R. parvicaudatus* (32saxIC, 27litHR), *C. lit. sax.*VIII (7saxIP, 26saxBP) *R. somateria* (10 nIR), *C. doricha* (DQ489708.1 *Cercaria doricha)*, *C. pythionike* (DQ489707.1 *Cercaria pythionike)*, *R. sternae* (KU563707.1 *Renicola sternae* voucher 3LF-2454), *R. pinguis* (KU563705.1 *Renicola pinguis* voucher 3LF-1166), *R. lari* (KU563706.1 *Renicola lari* voucher 3LF-2444), *R. sloanei*(KU563710.1 *Renicola sloanei* voucher 3LF-2530), *Microphallus* (HM584187.1 *Microphallus sp*. nov. 1 IBC-2010 isolate Mkur8B), *Fasciola* — AM850108.1 *Fasciola gigantica*.
